# Live cell visualization of the interactions between HIV-1 Gag and the cellular RNA-binding protein Staufen1

**DOI:** 10.1186/1742-4690-7-41

**Published:** 2010-05-10

**Authors:** Miroslav P Milev, Chris M Brown, Andrew J Mouland

**Affiliations:** 1 HIV-1 RNA Trafficking Laboratory, Lady Davis Institute for Medical Research-Sir Mortimer B. Davis Jewish General Hospital, 3755 Côte-Ste-Catherine Road., Montréal, H3T 1E2, Québec, Canada; 2 Department of Medicine, Division of Experimental Medicine, McGill University, Montreal, H3A 1A3, Quebec, Canada; 3 Department of Biochemistry, University of Otago, Dunedin 9001, New Zealand; 4 Department of Microbiology & Immunology, McGill University, Montreal, H3A 1A3, Quebec, Canada

## Abstract

**Background:**

Human immunodeficiency virus type 1 (HIV-1) uses cellular proteins and machinery to ensure transmission to uninfected cells. Although the host proteins involved in the transport of viral components toward the plasma membrane have been investigated, the dynamics of this process remain incompletely described. Previously we showed that the double-stranded (ds)RNA-binding protein, Staufen1 is found in the HIV-1 ribonucleoprotein (RNP) that contains the HIV-1 genomic RNA (vRNA), Gag and other host RNA-binding proteins in HIV-1-producing cells. Staufen1 interacts with the nucleocapsid domain (NC) domain of Gag and regulates Gag multimerization on membranes thereby modulating HIV-1 assembly. The formation of the HIV-1 RNP is dynamic and likely central to the fate of the vRNA during the late phase of the HIV-1 replication cycle.

**Results:**

Detailed molecular imaging of both the intracellular trafficking of virus components and of virus-host protein complexes is critical to enhance our understanding of factors that contribute to HIV-1 pathogenesis. In this work, we visualized the interactions between Gag and host proteins using bimolecular and trimolecular fluorescence complementation (BiFC and TriFC) analyses. These methods allow for the direct visualization of the localization of protein-protein and protein-protein-RNA interactions in live cells. We identified where the virus-host interactions between Gag and Staufen1 and Gag and IMP1 (also known as VICKZ1, IGF2BP1 and ZBP1) occur in cells. These virus-host interactions were not only detected in the cytoplasm, but were also found at cholesterol-enriched GM1-containing lipid raft plasma membrane domains. Importantly, Gag specifically recruited Staufen1 to the detergent insoluble membranes supporting a key function for this host factor during virus assembly. Notably, the TriFC experiments showed that Gag and Staufen1 actively recruited protein partners when tethered to mRNA.

**Conclusions:**

The present work characterizes the interaction sites of key components of the HIV-1 RNP (Gag, Staufen1 and IMP1), thereby bringing to light where HIV-1 recruits and co-opts RNA-binding proteins during virus assembly.

## Background

HIV-1 replication is characterized by multiple virus-host interactions that represent fundamental events enabling viral propagation. While Gag is central to assembly, numerous host proteins are also required for the generation of infectious HIV-1 particles [1]. The vRNA can both be translated to produce Gag and Gag-Pol or packaged into virions [2]. Gag selects the HIV-1 RNA genome (vRNA) for packaging in the cytoplasm. These events involve the regulated assembly of viral ribonucleoprotein (RNP) complexes. This is a prerequisite for successful retroviral vRNA trafficking from the nucleus into the cytoplasm, through the cytoplasm, and then into progeny virions at sites of assembly [3,4]. Importantly, recent studies show how vRNA transport mechanisms dictate to what extent both the vRNA is translated and to what efficiency Gag is assembled [5,6]. Studies also suggest that the host factors that interact with viral Gag and RNA might dictate intracellular trafficking events during viral egress (reviewed in [7]).

Initially Gag is synthesized as a precursor molecule, but is then cleaved to give rise to matrix (MA), capsid (CA), nucleocapsid (NC), a late domain (p6) plus two spacer peptides SP1 and SP2 during and following virus budding. The protein domains of Gag play distinct roles in the HIV-1 replication cycle (reviewed in [8]). During the assembly process MA targets Gag to membranes via its myristoylated highly basic N-terminus. Both the CA and the NC domain function in Gag-Gag multimerization [9-11]. Gag drives virion assembly and is sufficient for the organization, budding and release of virus-like particles (VLPs) from cells [12]. The association of Gag to membranes is essential for efficient viral replication. In fact, during viral egress, Gag rapidly associates to membranes that target to assembly sites [13,14] with the concerted activities of motor [15] and adaptor proteins [16-18]. Despite numerous studies, the contributions by cellular factors to the transport of Gag towards viral assembly platforms remain poorly understood. Recently, it was demonstrated that Gag preferentially mediates viral assembly at membrane lipid rafts. These are specific detergent-resistant microdomains implicated in multiple cellular processes (reviewed in [19]). HIV-1, like several other pathogens, also relies on membrane lipid rafts to complete its replication cycle (reviewed in [20]).

Previously, we demonstrated that Staufen1 interacts with Gag via the NC domain and influences Gag multimerization [21]. Staufen1's presence in the HIV-1 RNP that selectively contains the precursor Gag (pr55^Gag^) and the vRNA and not any other HIV-1 RNA species [22,23] and its eventual virion incorporation [24] promote the idea that Staufen1 has a regulatory role in HIV-1 assembly.

In the present study, we use BiFC analysis [25] to further characterize and visualize the interactions between Gag and Staufen1. Our results demonstrate that Staufen1 and Gag interact at both intracellular and plasma membrane compartments. In addition, we show that Staufen1 is recruited by Gag to the plasma membrane at lipid raft domains. TriFC analysis also showed that Staufen1 and Gag were able to recruit each other while bound to mRNA. Furthermore, when we depleted cells of Staufen1, multimerized Gag molecules were inefficiently localized to the plasma membrane, indicating that Staufen1 modulates the localization of the assembling Gag. This work provides new information on how HIV-1 co-opts cellular factors to ensure proper viral assembly.

## Results

### Bimolecular fluorescence complementation (BiFC) to visualize Gag-Staufen1 interactions in live mammalian cells

**Figure 1:**
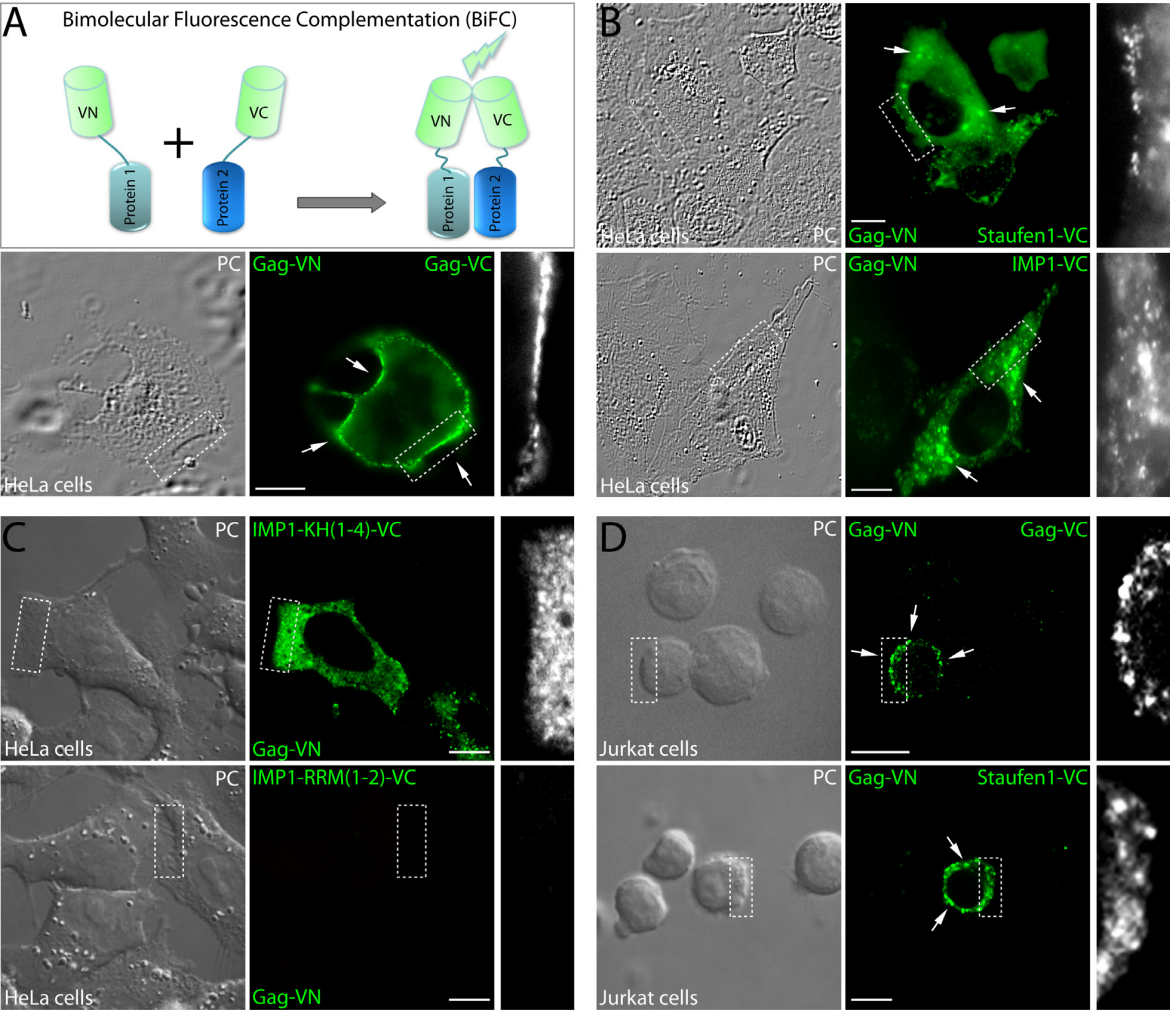
**Gag interactions with host proteins Staufen1 and IMP1 occur in the cytoplasm and at the plasma membrane of transfected HeLa and Jurkat T cells as determined by BiFC**. **(A) **Top - schematic representation of BiFC method. Bottom - Rev-dependent Gag-VN and Gag-VC were co-transfected with pCMV-Rev in HeLa cells. At 24 hr post-transfection, cells were imaged by laser scanning confocal microscopy to detect BiFC. The white arrows indicate plasma membrane concentrated accumulations of Gag-Gag BiFC signals. **(B) **Gag-VN and Staufen1-VC (top panels) or Gag-VN and IMP1-VC (bottom panels) interactions identified by BiFC. BiFC signals for these interacting pairs were mainly detected in the cytoplasm (indicated by white arrows) and at or near the plasma membrane. **(C) **Interactions between Gag-VN with IMP1-KH(1-4)-VC (top) and with IMP1-RRM(1-2)-VC (bottom) as determined by BiFC analysis. Evidence for interaction is demonstrated by a green fluorescence signal. **(D) **The interaction between Gag-VN and Gag-VC (top) or Gag-VN and Staufen1-VC (bottom) was determined by BiFC in Jurkat T cells. Magnified sections demonstrate details on the shapes of BiFC signals/complexes. The size bars are equal to 10 μm.

Recently, the relationship between Staufen1 and precursor Gag molecule (pr55^Gag^) was characterized. While Staufen1 is found predominantly in the cytoplasm at the endoplasmic reticulum [26]; Gag is localized in a punctate, non-uniform pattern throughout the cytoplasm and is enriched at the plasma membrane [13]. Here, we used the BiFC assay because it enables live cell visualization of protein-protein interactions. Moreover, it has proven to provide a reliable read-out of protein-protein interaction sites in several cell types and organisms [6,27-31]. As a starting point for this part of our research, we studied the interaction between Rev-dependent Gag proteins as depicted in Figure 1A (top) [6]. Gag multimerized and assembled with high efficiency as shown by strong green fluorescence signals due to Gag-VenusC (VC) and VenusN (VN) BiFC (Figure 1A, bottom panels). Gag-Gag multimerization occurred at the plasma membrane, and numerous Gag-Gag interaction events were also seen within the cytoplasm.

We then characterized the interaction between Gag and Staufen1. These proteins are known to interact in a RNA-independent manner [22] and are in close proximity (≈10 nm) as determined by bioluminescence resonance energy transfer experiments [21-23], thus we expected to observe BiFC; but in addition, we wanted to identify the interaction sites for this virus-host pair. We detected small and large robust BiFC signals in the cytoplasm. Furthermore, a close examination of cells revealed that the Staufen1 and Gag BiFC signals coincided with the plasma membrane periphery (Figure 1B, top panels), similar to what was found for Gag. This was observed in over 90% of cells (n > 300) exhibiting BiFC.

**Figure 2:**
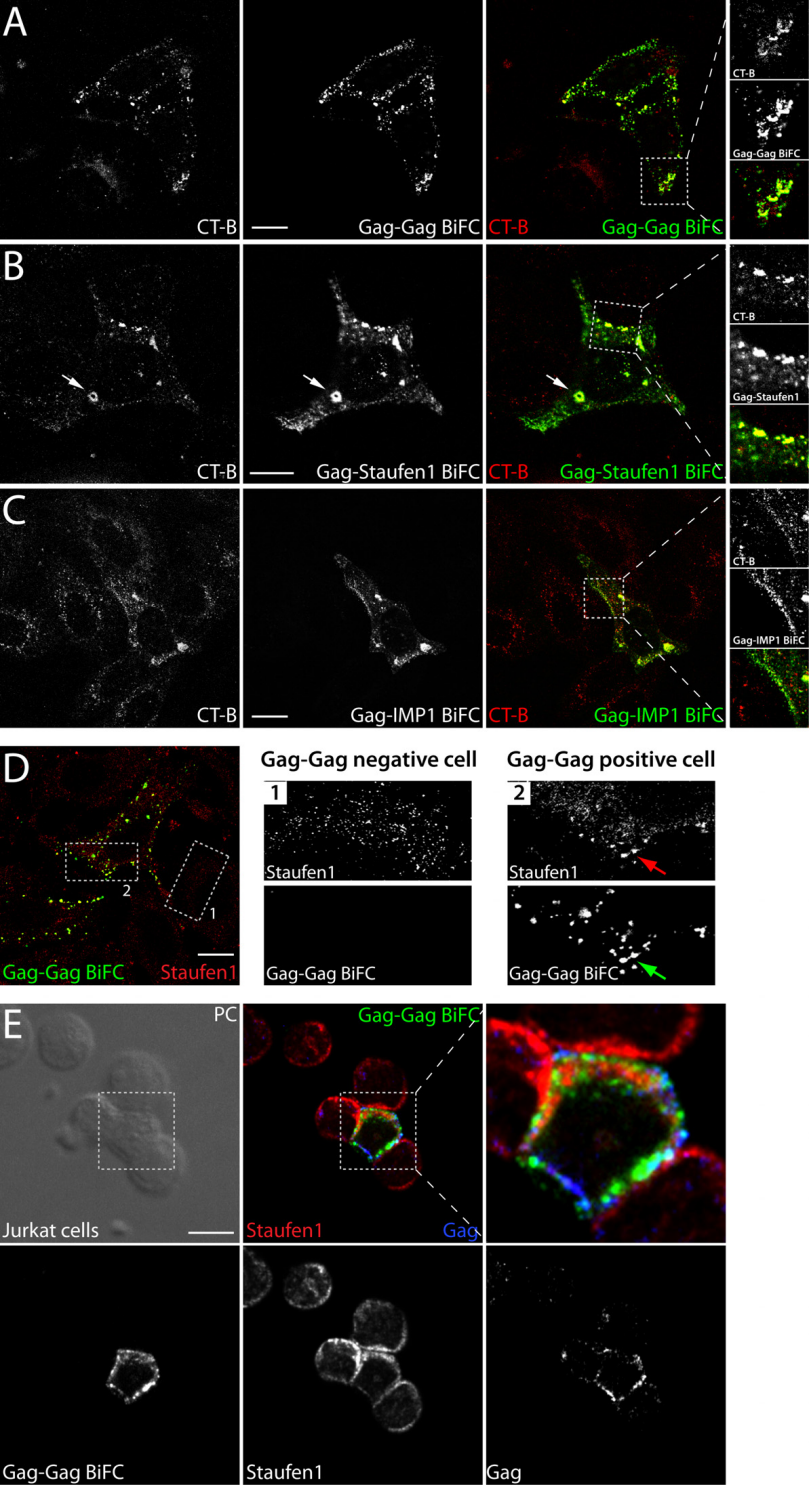
**Interactions between Gag and cellular proteins Staufen1 or IMP1 at GM1 containing lipid rafts on the plasma membrane as determined by BiFC**. **(A) **HeLa cells were co-transfected with pCMV-Rev and Rev-dependent Gag-VN and Gag-VC plasmids. At 24 hr post-transfection lipid raft staining in live cells was performed. Images were captured using laser scanning confocal microscopy to detect the co-localization patterns of oligomerizing Gag molecules and lipid raft microdomains (indicated by CT-B that binds the pentasaccharide chain of the raft marker protein, GM1). **(B) **Gag-VN/Staufen1-VC BiFC signals and CT-B staining in live cells. **(C) **Gag-VN/IMP1-VC BiFC signals and CT-B staining in live cells. **(D) **HeLa cells or **(E) **Jurkat T cells were co-transfected with pCMV-Rev and Rev-dependent Gag-VN and Gag-VC plasmids. At 24 hr post-transfection the cells were fixed in 4% paraformaldehyde (T cells were attached to poly-D-lysine coated coverslips before fixation), permeabilized in 0.2% Triton and stained for endogenous Staufen1 and p17 to detect Gag (in Jurkat T cells only; **(E)**, Gag is presented in blue). BiFC signals also identify Gag-Gag oligomers in fixed cells. Magnifications of cells on right show endogenous Staufen1 in Gag-Gag BiFC-negative (box 1) and positive (box 2) HeLa (D) or Jurkat T (E) cells. The size bars are equal to 10 μm.

We also performed BiFC to identify where Gag and Insulin like growth factor II mRNA binding protein (IMP1) interacted in cells. We chose IMP1 because it is a component of the Staufen1 RNP [32,33] and because IMP1 associates to Gag and is incorporated in HIV-1 [34,35]. The co-expression of Gag-VN and IMP1-VC generated intense BiFC signals predominantly in the cytoplasm (Figure 1B, bottom panels) with a detectable amount at the plasma membrane in some cells (not shown; see Figure 2C). IMP1-Gag exhibited a very specific and abundant interaction and shared some features with the interaction site that we identified for Staufen1-Gag including well defined cytoplasmic and plasma membrane foci. The Gag-binding domain in IMP1 was mapped to the four KH RNA-binding domains [35]. Therefore we performed BiFC analysis; and as expected, the IMP1-KH(1-4)-Gag interaction was maintained (Figure 1C, top panels) whereas the expression of IMP1-RRM(1-2), lacking the interaction domain, failed to complement with Gag in this assay (Figure 1C, bottom panels). A variety of other negative controls were performed. For example, the co-expression of bacteriophage coat protein MS2 fused to the VN moiety with either Gag-VC, Staufen1-VC or IMP1-VC did not produce BiFC signals in any cell, demonstrating the specificity of the method (Additional file 1: Figure S1-A). Furthermore, we expressed the BiFC Gag moieties along with pNL4.3 proviral DNA at a 1:5 molar ratio in order to demonstrate that the resulting BiFC signals are specific and not due to artifacts created by Gag-VC/VN overexpression or changes in the kinetics of viral particle assembly, consistent with an earlier report [36]. BiFC will only be positive in cells expressing pNL4.3 in the presence of Rev. Importantly, this experimental set up, that also includes the expression of the full complement of viral genes, leads to identical BiFC signals (Additional file 1: Figure S1-B). Finally, BiFC analyses were performed in Jurkat T cells, and again, robust Gag-Gag and Gag-Staufen1 BiFC signals were evident at the periphery of T cells (Figure 1D).

### Association of Gag and cellular factors at GM1-containing lipid rafts on the plasma membrane

Our earlier reports indicated that Staufen1 associates with vRNA and Gag in both cells and virus [22,24]. Our recent data suggest that this host protein modulates Gag multimerization on membranes [21]. Gag preferentially mediates viral assembly at specific sites on the plasma membrane called lipid raft microdomains [37,38] that are composed of cholesterol and sphingolipids, and contain several other components such as ganglioside GM1, glycophosphatidylinositol-anchored (GPI-anchored) proteins, tyrosine kinases of the Src family and others. Because the Gag and Staufen1 interaction occurs also on well defined plasma membrane structures (Figure 1B), we next determined the nature of these interaction domains using BiFC concomitant with live cell lipid raft staining. We transfected cells with Gag-VN and Gag-, Staufen1- or IMP1-VC BiFC constructs, and at 24 hr stained lipid rafts using AlexaFluor 594-labeled cholera toxin subunit B (CT-B) as described in Materials and Methods. As a reference condition for the association and multimerization of Gag on the plasma membrane, we again used Gag-VN and Gag-VC in BiFC (Figure 2A). We observed an almost complete co-localization of CT-B label and oligomerizing Gag, which is in accordance with previously published work [39-41]. Furthermore, BiFC between Gag-VN and Staufen1-VC followed by GM1 labeling revealed that a substantial part of these interactions also occurred at lipid rafts (Figure 2B). Likewise, a proportion of the Gag-IMP1 BiFC signals coincided with lipid raft domains, although as reported above, the plasma membrane localization was not as marked (Figure 2C).

Staufen1's abundance at the plasma membrane was puzzling since it is normally distributed in the cytoplasm co-localizing with the endoplasmic reticulum [26,42]. Therefore, to determine if Staufen1 is recruited by Gag to lipid raft domains, we co-transfected HeLa cells with the BiFC plasmid pair Gag-VN and Gag-VC, and at 24 hr post-transfection, we fixed and then stained the cells for endogenous Staufen1 (Figure 2D). The BiFC signals between Gag-VN/Gag-VC were observed at the plasma membrane and were preserved following fixation. Notably, abundant staining for endogenous Staufen1 coincided with the majority of the Gag BiFC signals in whole cells (Figure 2D, left panel) and in the expanded region on the right (Figure 2D, Gag-Gag positive cell) whereas in Gag-Gag negative cells, Staufen1 was dispersed in the cytoplasm. Thus, Staufen1 is recruited presumably by Gag to plasma membrane lipid rafts. Finally, we performed a similar analysis for endogenous Staufen1 in Jurkat T cells. Upon expression of Gag-VC and Gag-VN, endogenous Staufen1 coincided with Gag BiFC signals at cell-to-cell contact sites in Gag-expressing Jurkat T cells (Figure 2E).

We also observed Staufen1-Gag BiFC signals at intracellular domains marked by CT-B. These sites appeared to be vesicular in nature and were observed in ~75% of all cells examined (in >200 cells in 6 experiments; Figure 2B, white arrow). These sites of interaction with CT-B staining represent either rapidly internalized raft membrane domains or sites of raft biogenesis/synthesis [43,44]. To characterize them further, we performed time lapse imaging of live cells. The structures were mostly immobile, but several were dynamic showing characteristics of membranes that were capable of fusion, fission, detachment and subsequent trafficking towards the plasma membrane (Additional file 2: Figure S2). This result suggests that Gag passes through intracellular lipid raft membrane domains on its way to the plasma membrane.

### Biochemical fractionation of lipid rafts

**Figure 3:**
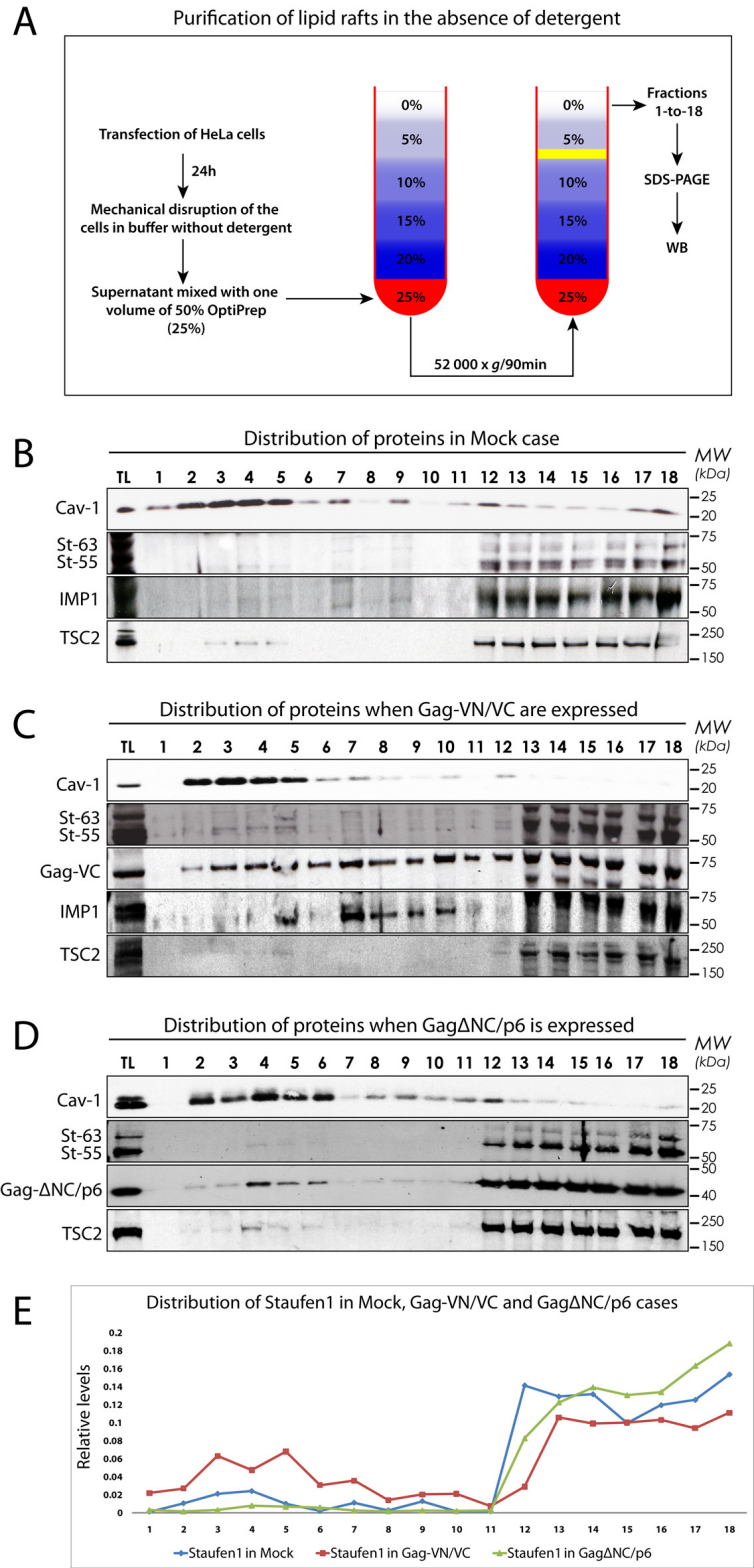
**Staufen1 co-fractionates with Gag in lipid rafts**. **(A) **A detergent-free method for the isolation and fractionation of lipid rafts. HeLa cells were mock transfected **(B) **or co-transfected with pCMV-Rev and Rev-dependent Gag-VN and Gag-VC **(C) **or Rev-dependent GagΔNC/p6 **(D)**. At 24 hr post-transfection, cells were harvested and fractionated on Optiprep gradients: lipid rafts fractionated in fractions #2-6 and non-membrane associated proteins in the bottom fractions. Western blotting identified Caveolin-1 (Cav-1), Staufen1 (two isoforms: St-55, St-63), IMP1, TSC2, Gag-VC (in C) and p24 (to detect GagΔNC/p6 in (D)) in each fraction. **(E) **The relative quantities of Staufen1 in each fraction were measured using ImageJ software (NIH) (the sum of all fractions per condition = 1.0).

We performed a detergent-free membrane flotation assay to purify lipid rafts with the advantages that fewer insoluble aggregates form, and the purifications are met with less contamination from non-raft cellular membranes ([45]; Figure 3A). We either mock transfected HeLa cells or co-transfected them with Gag-VN and Gag-VC plasmids to reproduce our BiFC conditions above. Alternatively, cells were transfected with a Rev-dependent GagΔNC/p6 construct [46] as a negative virus assembly control. At 24 hr post-transfection cells were lysed, washed and processed for fractionation. Eighteen fractions from each gradient were probed for the raft marker Caveolin-1 and for Staufen1, IMP1 and Tuberin (TSC2). Gag was detected with either an anti-GFP (recognizing VC of Gag-VC; Figure 3C) or with an anti-p24 (Figure 3D) antibody. Lipid rafts and associated proteins such as Caveolin-1 accumulated principally in fractions #2 to #6 in all conditions (mock and in the presence of Gag or truncated Gag proteins). The cytoplasmic protein TSC2 principally sedimented to fractions #12 to #18 (Figure 3B-D, representing non-membrane fractions), but small amounts were detected in association with rafts in the upper fractions as reported [47]. In mock conditions, a fraction of Staufen1 sedimented to the lipid rafts (≈6.5% of total Staufen1; Figure 3B &3E). In the presence of Gag however, a notable three-fold increase of Staufen1 (≈19%) fractionated to lipid raft fractions (Figure 3C &3E) with ≈14% of total Gag sedimenting within these fractions. In addition, a shift of IMP1 was observed within the gradient when Gag was expressed. Approximately 10% (vs ≈6% in mock conditions) and ≈31% (vs ≈5% in mock conditions) of IMP1 was found in lipid raft and intermediary fractions (#7-#10), respectively. This was consistent with our imaging data that detected a small proportion of IMP1 in the lipid rafts in Gag-expressing cells (Figures 1 &2). Of interest is the observation that IMP1 overexpression disrupts the association of mature Gag products on membranes [35]; therefore, the abundance of IMP1 on lipid raft domains might be underrepresented. LAMP-3/CD63 reactivity co-sedimented to these intermediary fractions (M.P.M. & A.J.M., data not shown) and further study of these membrane domains will be necessary. As a control we expressed the assembly defective GagΔNC/p6 which can not bind to several host proteins like Staufen1, IMP1 and HP68 (ABCE1), for example [22,35,46]. Neither GagΔNC/p6 nor Staufen1 sedimented to any great extent to the lipid raft fractions (Figure 3D) indicating a dependence on Gag for the enhanced recruitment of Staufen1 to lipid rafts. These biochemical data reflect our imaging data that showed a recruitment of Staufen1 to lipid raft microdomains in the majority of Gag-transfected cells (Figure 2B &2D).

### Depletion of membrane cholesterol by hydroxy-propyl-β-cyclodextrin (HβCD) reduces Gag-Gag and Gag-Staufen1 membrane BiFC

**Figure 4:**
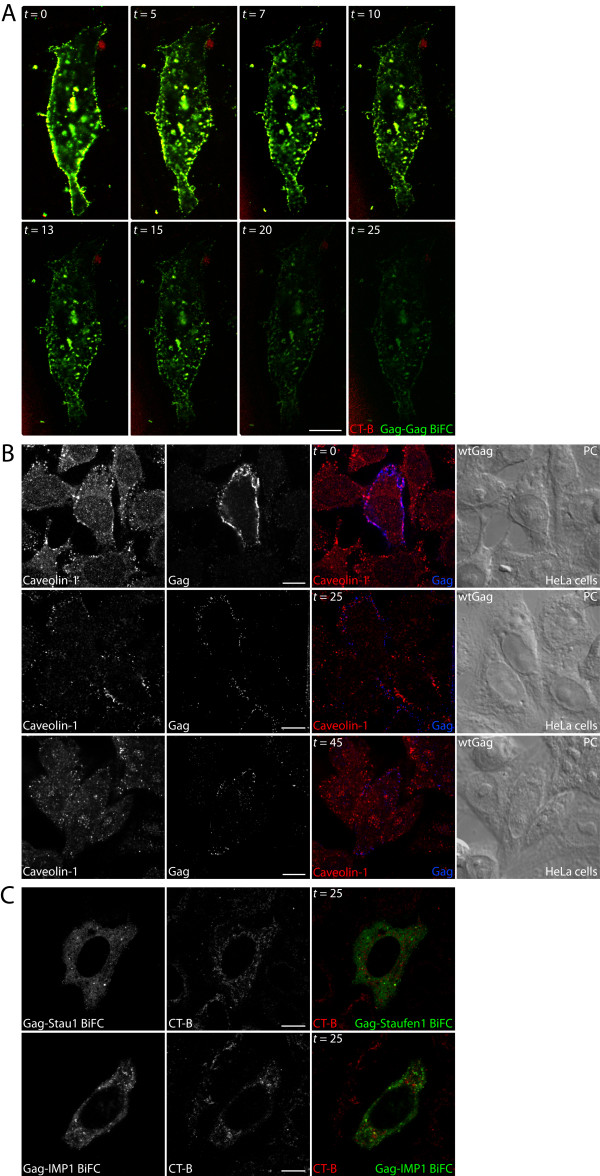
**Time-dependent depletion of cholesterol from lipid rafts leads to the disruption of Gag-Gag, Gag-Staufen1 or Gag-IMP1 BiFC**. **(A) **HeLa cells were co-transfected with pCMV-Rev, Gag-VN and Gag-VC. At 24 hr post-transfection the cells were stained with the Vybrant Lipid Raft Labeling Kit and treated with cholesterol disrupting drug HβCD (final concentration 30 mM). Pictures were taken at the indicated times post-HβCD treatment. **(B) **HeLa cells were co-transfected with pCMV-Rev and the Rev-dependent Gag [46] and at 24 hr post-transfection were treated with HβCD for different periods of time (as indicated, for up to 45 min). The cells were then fixed, stained for Caveolin-1 and Gag and visualized by laser scanning confocal microscopy. **(C) **Hela cells were co-transfected with pCMV-Rev and either Gag-VN and Staufen1-VC or Gag-VN (top panels) and IMP1-VC (lower panels). At 24 hr post-transfection lipid rafts were identified in live cells using the Vybrant Lipid Raft Labeling and treated with HβCD for up to 25 min. The BiFC signals were determined at *t *= 0 (not shown) and at the latest time point of *t *= 25 min. The size bars are equal to 10 μm.

Since cholesterol is essential for lipid raft structure and function, its depletion should disrupt BiFC signals occurring at these sites. Indeed, cholesterol depletion from the rafts reduces the total amount of associated Gag and more specifically the presence of higher-order Gag multimers on the plasma membrane [38]. Thus, to confirm that the plasma membrane domains where we find Gag-Staufen1 BiFC are lipid rafts, we depleted cholesterol from membranes using HβCD. At various time points between 0 and 25 minutes, lipid rafts were detected by CT-B staining; and the BiFC signals were imaged by laser scanning confocal microscopy. Multimerized Gag demonstrated strong association with GM1-containing lipid microdomains before addition of HβCD (Figure 4A, *t *= 0 min). We then perfused cells with HβCD and collected images from live cells at time points thereafter. Time-lapse imaging revealed a significant decrease in CT-B staining at all time points after 15 minutes indicating that HβCD was effective at disrupting lipid rafts. The BiFC signals for Gag-Gag also dramatically decreased over time (Figure 4A). The decreases in Gag-Gag and Gag-Staufen1 BiFC signals were likely due to the disruption of plasma membrane assembly domains thereby preventing both the accumulation of Gag and the bimolecular interactions. Because we took multiple laser scans of the same cells, we attempted to rule out any effect photobleaching might have in the BiFC and CT-B signals captured at the later time points. We therefore transfected cells with a Rev-dependent Gag construct and at 24 hr treated them with HβCD over an extended period of time (0-45 min). Cells were subsequently fixed, and immunofluorescence was performed to obtain snapshots of the distributions of Gag and of the raft marker protein, Caveolin-1 (Figure 4B). Indeed, both Caveolin-1 and Gag staining were diminished over the course of this experiment. Therefore, we can conclude that photobleaching is not significant in these experiments, and also that the scaffold for interactions between Gag and host proteins is disrupted by cholesterol depletion. Finally, whereas the BiFC signals for both the Staufen1-Gag and IMP1-Gag interactions were observed at time 0 (data not shown but refer to Figures 2 and 3), these substantially decreased over time following HβCD treatment indicating that intact lipid rafts contribute to these bimolecular interactions (Figure 4C).

### Effects of modulating Staufen1 levels on the distribution of Gag-Gag BiFC complexes

**Figure 5:**
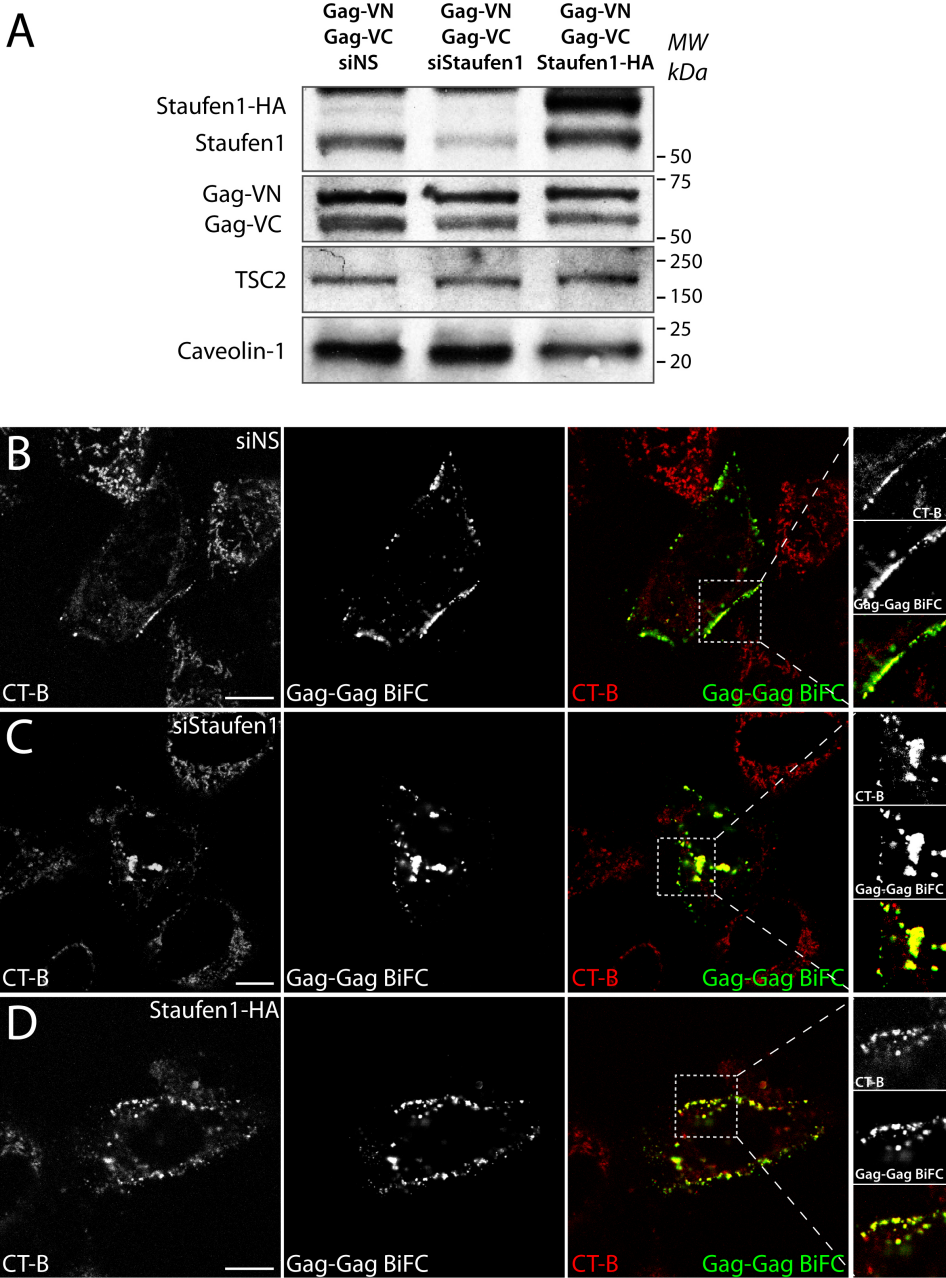
**Staufen1 depletion decreases plasma membrane-associated Gag and results in intracellular clustering of Gag-Gag BiFC signals**. HeLa cells were co-transfected with pCMV-Rev, Gag-VN and Gag-VC plasmids with control non-silencing siRNA (siNS), Staufen1 siRNA (siStaufen1) or Staufen1-HA. At 24 hr post-transfection cells were harvested for western blotting for Staufen1, Gag, Caveolin-1 and TSC2 (as loading controls) **(A) **or stained for lipid rafts in live cells. BiFC signals and lipid raft (CT-B) staining were captured by laser scanning confocal microscopy in cells transfected with siNS **(B)**, siStaufen1 **(C) **or Staufen1-HA **(D)**. Black and white images of lipid rafts (CT-B) and Gag-Gag BiFC signals and merged colour representations are shown. The insets are magnifications of boxed areas. The size bars are equal to 10 μm.

To characterize the role of Staufen1 in the trafficking and formation of Gag-Gag assembly complexes in live cells, we depleted or overexpressed Staufen1 using siStaufen1 or a Staufen1-HA cDNA, respectively [22,24]. The depletion of Staufen1 was efficient and reduced expression levels to less than 1/6 while the over-expression increased cellular Staufen1 levels approximately 6-fold (Figure 5A). In cells transfected with a control siRNA (siNS) co-expressing Gag-VN and Gag-VC, Gag BiFC was found at the plasma membrane and in discrete cytoplasmic domains as shown earlier (Figure 5B). However, in Staufen1-depleted cells, in addition to the Gag-Gag BiFC signals observed at the plasma membrane, strong signals were observed at cytoplasmic juxtanuclear regions (Figure 5C). When Staufen1-HA was over-expressed, we observed that the Gag-Gag BiFC punctae were abundant and well defined, and we could not detect any marked changes in plasma membrane association of Gag-Gag BiFC compared to the control siNS condition (Figure 5D). Neither the depletion nor the over-expression of Staufen1 caused any significant redistribution of ABCE1, a host factor that interacts with NC domain of Gag and is involved in assembly [46,48], in relationship to the localization of Gag-Gag BiFC or *gag *RNA signals (Additional file 3: Figure S3). Likewise, the depletion of the Staufen1^63 kDa ^isoform alone [49] or the depletion of UPF1 [32] did not result in intracellular Gag BiFC signals (M.P.M., Lara Ajamian and A.J.M., data not shown).

**Figure 6:**
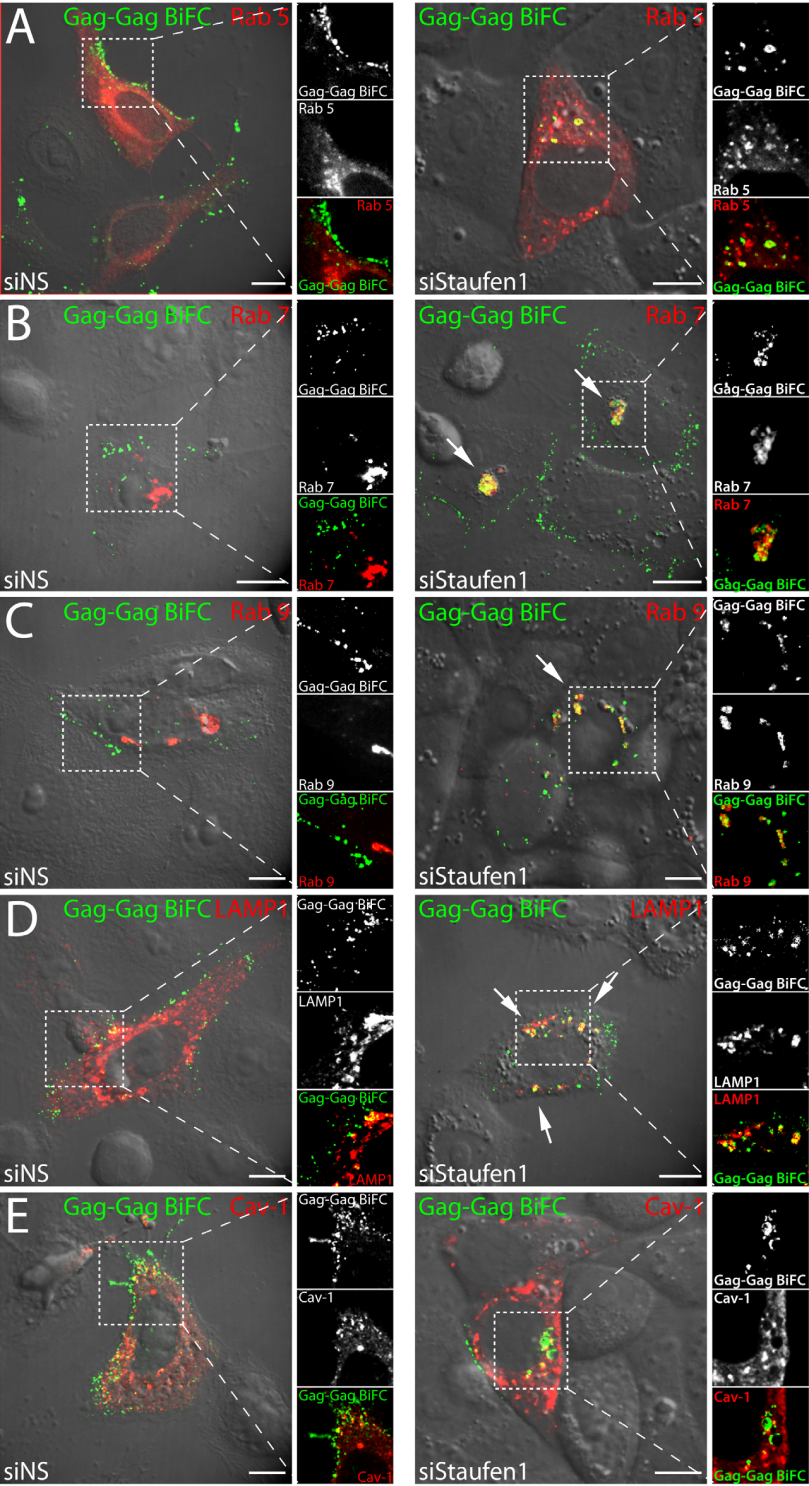
**Gag localizes near Rab7-, Rab9- and LAMP1-containing membranes at cytoplasmic and juxtanuclear sites in Staufen1-depleted cells**. HeLa cells were transfected with pCMV-Rev, Gag-VN and Gag-VC with either siNS or siStaufen1 siRNAs and one of the following plasmids: **(A) **Rab5-RFP, **(B) **Rab7-RFP, **(C) **Rab9-RFP, **(D) **LAMP1-RFP or **(E) **Caveolin-1-RFP. At 24 hr post-transfection, the distributions of Gag-Gag BiFC and RFP fusion proteins were visualized in live cells by laser scanning confocal microscopy. The insets show zoomed boxed regions of cells to demonstrate the levels of co-localization of Gag-Gag BiFC signals with either of membrane marker proteins. White arrows identify Gag-Gag BiFC aggregates. The size bars are equal to 10 μm.

We noticed earlier that Gag-Gag BiFC occurred on sometimes circular, membrane-like structures. Moreover, Gag, Staufen1 and vRNA traffic on endosomal membranes and in a manner that is dependent on endosomal vesicle positioning [13,14]. Therefore to determine the nature of the Gag structures, we co-expressed RFP fusion marker proteins Rab5 (early endosomes), Rab7 (late endosomes), Rab9 (Golgi/endoplasmic reticulum), LAMP1 (late endosomes) and Caveolin-1 (lipid rafts/caveosomes) in Staufen1-depleted cells. This analysis revealed that the Gag-Gag BiFC signals were on membranes that bore characteristics of endosomal membranes/vesicles, consistent with recent work ([13,14]; Figure 6). Specifically, the Gag-Gag BiFC multimers that coalesced intracellularly upon Staufen1 depletion coincided to varying extents with late endosomal membrane components LAMP-1-, Rab7- and on Rab9-containing membranes. While Staufen1 depletion did not influence the patterns of Rab7, Rab5, endoplasmic reticulum or Golgi staining (M.P.M. and A.J.M., data not shown), we nevertheless attempted to identify the origin of the Gag BiFC signals in Staufen1-depleted cells. To do this, we overexpressed two *trans*-dominant negative (TDN) proteins that efficiently block endocytosis at various steps as described [13]. These include Eps15-TDN-GFP and Rab7-TDN-GFP. These have been shown to block early endocytic events as shown by a block to Transferrin receptor recycling. Identical distributions of the Gag (expressed here as Rev-dependent Gag only [46]) signals were obtained when clathrin-dependent and clathrin-independent (M.P.M. and A.J.M., data not shown) endocytosis was blocked in Staufen1-depleted cells (Additional file 4: Figure S4 and data not shown). Therefore, we propose that the localization of the BiFC signals obtained in Staufen1-depleted cells was unlikely to be due to endocytosed Gag-Gag multimers (see Discussion).

### Trimolecular fluorescence complementation (TriFC) to visualize specific Gag- RNA interactions in living mammalian cells

**Figure 7:**
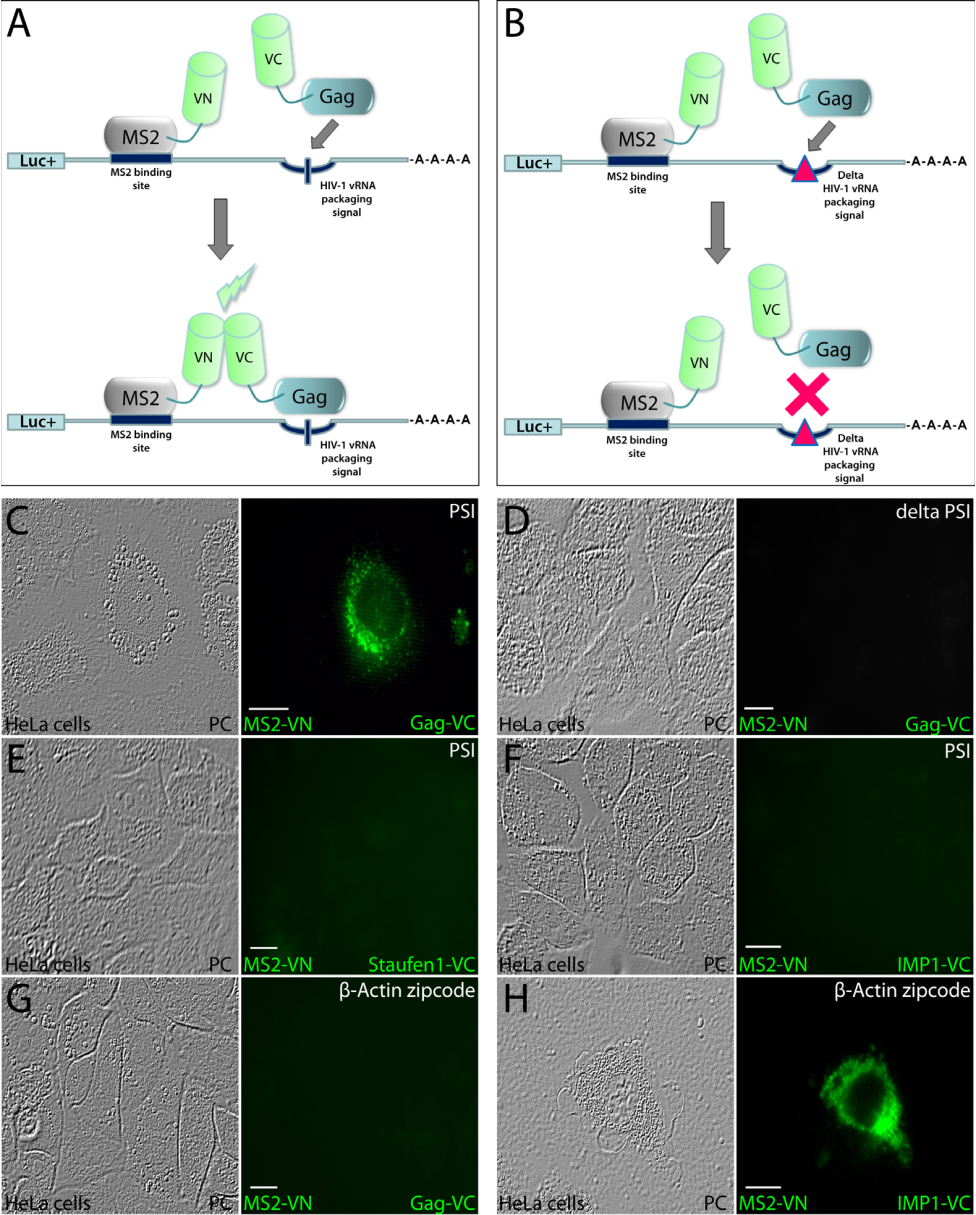
**TriFC analysis for studying RNA-protein interactions in live cells**. **(A) **Depiction of the TriFC analysis employed in the study. The mRNA-reporter molecule contains HIV-1 vRNA packaging signal *psi *in close proximity to the integrated MS2 RNA-binding site (MS2BS). MS2-VN binds MS2BS to tether to the mRNA molecule. The C-terminal moiety of Venus (VC) is expressed as hGag-VC fusion and binding of hGag-VC to the RNA packaging sequence (*psi *domain) will bring both VN and VC Venus moieties in close enough proximity to generate a fluorescent signal by VN-VC complementation. **(B) **A 19-basepair deletion from SL3 of *psi *prevents the binding between hGag-VC and *psi *RNA and does not yield fluorescence complementation. **(C) **HeLa cells were grown on coverslips and transfected with pGL3MS2site-*psi*, MS2-VN and Gag-VN. 24 hr later laser scanning confocal microscopy was used to assess TriFC. Bright fluorescence signals in cytoplasmic punctae were detected indicating that the interaction between hGag-VC and the *psi *RNA domain occurred. This condition represented a positive control for the TriFC system employed here. TriFC signals were not detected when HeLa cells were transfected with the mutated *psi *reporter (pGL3MS2site-Delta-*psi*) **(D)**. In order to determine the specificity of the TriFC assay, HeLa cells were co-transfected with plasmids expressing either Staufen1-VC **(E) **or IMP1-VC **(F) **along with MS2-VN and pGL3MS2site-*psi*. TriFC was absent in all cells examined indicating that Staufen1 and IMP1 do not bind the *psi *RNA. In parallel experiments, Gag did not associate with mRNA reporter bearing β-Actin zipcode sequence (pGL3-βActin zipcode) **(G) **whereas IMP1 did **(H)**, as expected [33]. Phase contrast images are shown on the left of each panel in (**C**)-(**H**). The size bars are equal to 10 μm.

Gag-Gag and Gag-Staufen1 interactions as visualized by BiFC were found consistently in the cytoplasm and at plasma membrane lipid raft domains. Because both Gag and Staufen1 bind mRNAs, we therefore explored how the association to mRNA would influence the Gag-Gag and Gag-Staufen1 associations. To do this, we employed TriFC, a method that allows the detection of protein-RNA or protein-protein-RNA interactions in live cells [33]. To validate this assay we took advantage of the well characterized Gag-vRNA association mediated by the *psi *RNA packaging signal sequence. Thus, we generated constructs that expressed mRNAs containing either the complete packaging signal *psi *(pGL3MS2site-*psi*; including SL2 and SL3 sequences), or delta *psi *domain in which 19 nucleotides are deleted from SL3 (pGL3MS2site-Delta-*psi*; Table 1; [50]). The mRNA reporters, based on pGL3MS2 site/basic [33], were also designed to contain an MS2 binding site that provides an efficient tethering of MS2-VN fusion protein. The K_D _of this interaction falls in the nanomolar range [51], and it likely occurs rapidly and is stable. As shown in Figure 7A in order for the successful complementation of both VN and VC fragments the simultaneous expression of three plasmids is required. In the first case the VN is expressed as a fusion MS2-VN protein and can tether to the RNA reporter in proximity to the *psi *sequence (Figure 7A). hGag-VC will interact with the *psi *domain of the reporter to allow for fluorescence complementation (Figure 7A); or when the *psi *is mutated, fluorescence complementation will not occur (Figure 7B). Using this assay, we found intense TriFC signals in the juxtanuclear region and cytoplasm (Figure 7C). In contrast, we did not detect TriFC when the mRNA reporter harboring the mutated *psi *(delta *psi*) was expressed (Figure 7D), and this was found in at least 5 experiments and having examined >400 viable cells. This result was in accordance with our expectation because packaging into virions of the virus-specific RNA harboring this deletion was less than 2% of that of the wild-type virus [50]. These findings indicate that the association of Gag with mRNA reporter molecules is sufficient to sequester them into granules that bear hallmark appearance of RNP containing proteins and mRNA [33]. The experiments demonstrated the biological relevance and specificity of the interactions between Gag and the HIV-1 vRNA *psi *packaging domain detected by TriFC. When full-length Venus was expressed in the context of the fusion MS2-Venus and co-expressed with the reporter mRNA bearing MS2 binding domains, a uniformly distributed fluorescence signal was obtained throughout the cell, in contrast to what we have obtained above (Additional file 5: Figure S5). Additional negative controls included the expression of a *psi *mRNA reporter (pGL3MS2site-Delta-*psi*), the expression of Staufen1- or IMP1-VC fusion proteins (Figure 7E &7F) or the exclusion of the VN or VC expression vectors in the transfections (M.P.M. and A.J.M., data not shown). We also expressed an mRNA reporter that contains the β-Actin zipcode RNA sequence, and we did not detect fluorescence complementation (Figure 7G), whereas a strong fluorescence signal was obtained using the cognate binding protein IMP1-VC (Figure 7H; [33]). Because Gag expression is Rev-independent and may traffic aberrantly in this set of experiments, we modified the TriFC assay and employed a Rev-dependent Gag expressor. We co-expressed the luciferase mRNA reporter harboring the *psi *domain (or a mutated form as described above), MS2-VN to tether to the MS2 RNA loops, Staufen1-VC, a Rev-dependent Gag expressor [46] and pCMV-Rev as depicted (Additional file 6: Figure S6-A). In this setup Gag must be brought in proximity to the RNA in order to recruit Staufen1-VC to generate a TriFC signal. Indeed, robust TriFC signals were obtained in cells. The removal of MS2-VN, mutation of *psi *RNA and the expression of Rev-dependent GagΔNC/p6 ([46]; Additional file 6: Figure S6-B) abrogated the TriFC signals in all cells examined demonstrating the specificity of the assay.

**Table 1:** List of plasmids used in this study.

Plasmid name	Source or reference
pMS2-VenusFL	[33]

pMS2-VN	[33]

pMS2-Staufen1	This study

pMS2-hGag	This study

pIMP1-VC	[33]

pIMP1-KH(1-4)-VC	[33]

pIMP1-RRM(1-2)-VC	[33]

pStaufen1-VC	This study

pStaufen1-HA	[24]

phGag-VC	This study

pMS2-Gag(C36S)	This study

pGag-VN	[6]

pGag-VC	[6]

pCMV-Rev	NIH

pSVGagRRE-R	[70]

pGag-ΔNC/p6(Tr361)	[46]

pGL3MS2 site/basic	[33]

pGL3-βActin zipcode	[33]

pGL3MS2site-*psi*	This study

pGL3MS2site-Delta-*psi*	This study

pEps15-TDN-GFP	[13]

pRab5-TDN-GFP	[13]

mRFP-Rab5	Addgene plasmid 14437, [71]

dsRed-rab7	Addgene plasmid 12661, [72]

dsRed-rab9 WT	Addgene plasmid 12677, [72]

Cav1-mRFP	Addgene plasmid 14434, [73]

LAMP1-RFP	Addgene plasmid 1817, [74]

### TriFC visualization of protein-protein recruitment and interactions on mRNA template

**Figure 8:**
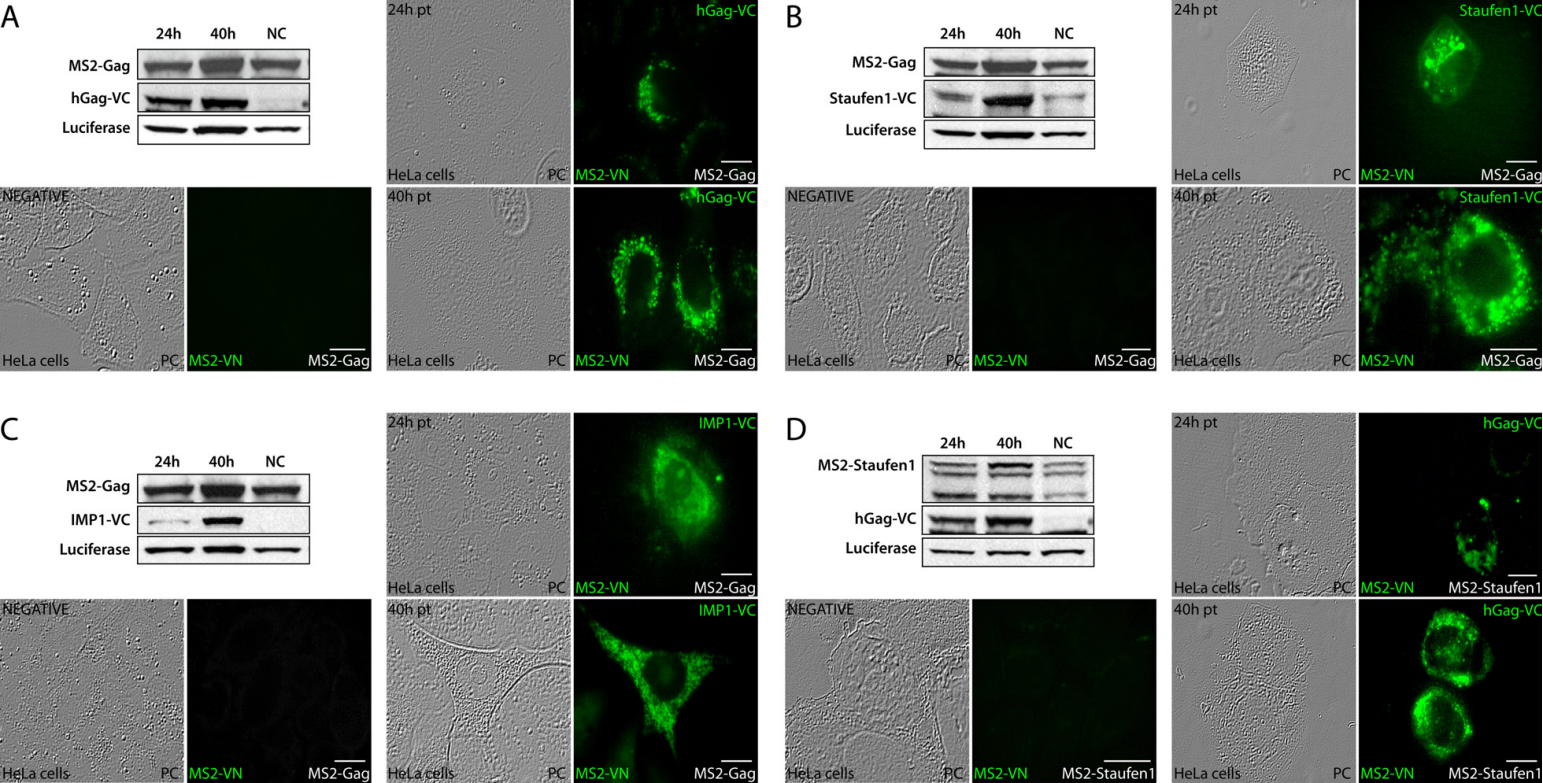
**Gag recruits host proteins Staufen1 and IMP1 while tethered to mRNA**. HeLa cells were co-transfected with mRNA-reporter pGL3-basic/site, MS2-VN, MS2-Gag or MS2-Staufen1 and hGag-VC, Staufen1-VC or IMP-VC as indicated. TriFC analysis was performed at 24 and 40 hr post-transfection. MS2 RNA-tethered hGag recruits Gag **(A)**, Staufen1 **(B) **and IMP1 **(C) **to generate TriFC signals in cells. MS2 RNA-tethered Staufen1 recruits hGag **(D)**. Western blotting analysis for Staufen1, p24 (to identify Gag), and GFP (that recognizes C terminal part of Venus) and Luciferase (reporter mRNA, pGL3-basic/site expressed Luciferase) confirmed expression of DNA constructs. Negative controls included transfections that omitted either the VC fusion proteins **(**bottom left in panels **(A)-(D)) **or the bridging MS2 molecule (MS2-hGag, MS2-Gag(C36S) or MS2-Staufen1; data not shown). The size bars are equal to 10 μm.

Using TriFC, we next evaluated whether Gag, while tethered to the mRNA, could recruit Staufen1 and other host proteins. MS2-Staufen1, MS2-hGag and MS2-Gag(C36S) expression constructs were created for this purpose. When mRNA reporters are co-expressed with MS2-VN and MS2-fusion proteins, these molecules will tether to the same MS2 RNA-binding site (MS2BS) on the mRNA. This is a native property of the bacteriophage coat protein that binds the MS2BS hairpin as a dimer [52,53]. In addition, in this system we expressed a second protein of interest as a fusion with VC. When MS2-hGag was co-tethered with MS2-VN to the mRNA reporter without any additional protein binding domains [33], hGag-VC was recruited to the mRNA as evidenced by bright TriFC signals (Figure 8A). At the later time point (40 hr), the punctae were larger, more abundant and were better defined. Nevertheless, the signal intensity and abundance of TriFC signals generally correlated with protein expression levels (Figure 8A). mRNA-tethered MS2-hGag recruited both Staufen1-VC and IMP1-VC (Figure 8B &8C). TriFC signals had apparent sizes ranging from 0.2 to 1.25 μm in diameter and were distributed in the cytoplasm of host cells, similar to what we found for hGag proteins (see Discussion). Because NC is the interacting domain for APOBEC3G [54], Staufen1 [22], and IMP1 [35], we tethered MS2-Gag(C36S) along with MS2-VN and assessed the level of interaction with Staufen1-VC. We did not detect fluorescence complementation in the cells (M.P.M. & A.J.M., data not shown).

When we tethered MS2-VN and MS2-Staufen1 to the mRNA via MS2BS and expressed Gag-VC, TriFC was readily detected, indicating that Staufen1, when bound to mRNA, recruits Gag (Figure 8D). These results demonstrate that Gag potentially recruits cellular factors while bound to an mRNA; and likewise, RNA-binding host factors can recruit Gag to mRNA. The results also support the notion that the HIV-1 RNP, containing at its core the precursor Gag and the genomic RNA, will selectively engage cellular factors such as Staufen1 and IMP1 during assembly.

## Discussion

### Gag-Staufen1 interactions in living cells

Staufen1 was previously found as a component of HIV-1 particles [24,55]. We have uncovered additional roles for this host factor including one in promoting Gag multimerization and assembly and another in the selection of vRNA for encapsidation [24,49]. In the present study, we extend our understanding of Staufen1's role in HIV-1 replication by characterizing its interactions with Gag using two powerful, live cell fluorescence complementation techniques. We demonstrate that Staufen1 interacts with Gag, that Staufen1 recruits Gag when it is bound to mRNA and likewise, Gag recruits Staufen1 when bound to the cognate *psi *packaging signal. The results highlight Staufen1's involvement in the HIV-1 RNP in the assembly of HIV-1.

### Recruitment of Staufen1 by Gag to lipid rafts and to mRNA

Our earlier work demonstrated that when we modulated Staufen1 levels in cells, Gag-Gag interactions increased, and these accumulated in detergent-resistant complexes [21]. Here, we used BiFC to evaluate where Staufen1 and Gag interact. This approach, which employs native Gag sequences, has become increasingly popular to visualize Gag in cells [56]. While Staufen1 and Gag are shown to associate in cytoplasmic compartments, our results also reveal that these interactions occur on membranes and at the plasma membrane where Gag multimerizes during assembly [6,21]. Whereas Staufen1 is usually found to localize on reticulotubular structures and the endoplasmic reticulum [26], we show that Staufen1 is recruited from the cytoplasm to lipid rafts at the plasma membrane where it interacts with Gag. Consistently, Staufen1 co-localizes with Gag and vRNA at this location [49], and the distribution of the Staufen1-interacting partner, UPF1, also shuttles to the plasma membrane domain in HIV-1 expressing cells [32]. Here, Gag expression alone appears to be the driving force behind Staufen1 recruitment to GM1-positive plasma membrane domains that serve as the main platforms for viral assembly [57]. Gag's ability to recruit host factors and bind *psi *RNA concomitantly, via the same protein domain (NC), reveals a rather multifaceted property of Gag and further strengthens the implication of Staufen1 in HIV-1 assembly [21]. Furthermore, time-lapse confocal imaging showed that Gag-Staufen1 foci are mobile and dynamic and are able to merge with similar structures and separate over time into smaller particles that traffic to and anchor at the plasma membrane (Additional file 2: Figure S2). Consistently, distinct populations of Gag and Staufen1 (and the vRNA) traffic on endosomal membranes in cells [13,14]. Moreover, the Gag-vRNA interactions as well intermediary Gag assembly domains have been found in juxtanuclear domains [15,58-60]. Thus, we propose that Staufen1 is hijacked by Gag shortly after its synthesis in order to assist in trafficking and assembly.

There are potentially two caveats with respect to the TriFc method used here. First is the use of codon-optimized hGag, that when expressed, could result in deviations in transport and assembly. Nevertheless, several recent studies have utilized Rev-independent hGag expressors to uncover new information on intracellular Gag trafficking and Gag interacting partners [6,29,56]. Furthermore, we show that by employing a Rev-dependent Gag expressor in a modified TriFC analyses nearly identical TriFC signals were obtained, revealing that codon-optimization of hGag did not have a significant bearing on the results. The second caveat is the possibility for an alternative interpretation that includes the order in which the protein-protein and protein-RNA interactions occur. We are claiming that either Gag or Staufen1 recruits the other partner while bound to mRNA, and this is supported by BiFC and endogenous Staufen1 staining (Figure 2). However, the bimolecular interaction between Gag and Gag and that found between Staufen1 and Gag could represent the initial event after which the bimolecular complex co-traffics to the mRNA substrate. Even though the K_D _for MS2-MS2BS interaction is in the low nanomolar range, further work will be necessary in order to confidently differentiate between some of these possibilities.

### Involvement of Staufen1 in the anterograde trafficking of Gag

We recently showed that a population of Staufen1 associates along with Gag and vRNA on endosomal membranes [13]. Likewise, RNPs translocate within cells by making use of machineries that direct traffic of cellular membranes and vesicles (reviewed in [61]). In this study we wanted to characterize the possible roles of Staufen1 in the transport and distribution of multimerizing Gag-Gag that we can readily visualize in live cells. To resolve this, we modulated Staufen1 expression levels in cells that expressed Gag-VN/Gag-VC. When we depleted Staufen1, Gag-Gag BiFC signals were found at the plasma membrane but also in the cytoplasm at juxtanuclear regions on what appeared to be endosomal membrane vesicles. We suspect that the Gag-Gag BiFC signals are due to coalescing HIV-1 RNPs that tether to endosomal membrane populations that may not traffic properly. These were identified in earlier work [13] and were found to form in an HIV-1-dependent manner containing Staufen1, Gag (and Gag multimers) and vRNA reactivity later named Staufen1 HIV-1-dependent ribonucleoproteins (SHRNPs; [49]). The data shown here are consistent with the enhanced Gag-Gag multimerization in Staufen1-depleted cells that we found using another biophysical technique (bioluminescence resonance energy transfer) [21], but also reveal some of the principal locations of these events (at juxtanuclear and raft domains) [22,49]. It is possible that, during viral egress, Gag mediates the formation of its own mixed type of membranes bearing several endosomal marker proteins similar to those that other viruses engineer [62]. Of interest are data from recently published work in which the accumulation of HIV-1 Gag and cholesterol-enriched membranes was observed in juxtanuclear late endosomal compartments in Niemann-Pick type C1-deficient cells [63]. Furthermore, the targeted depletion of the suppressor of cytokine signalling 1 reduced the transport of Gag to the plasma membrane, leading to accumulations of it near the nucleus [64]. In addition, strong co-localization between another late endosomal marker, CD63, and Gag was observed as a result of Rab9 depletion [65]. These domains appear to be important for steps in assembly including Gag-Gag and Gag-vRNA associations [13,58,59], vRNA encapsidation [49] and Gag trafficking [60,66] and in one case, for Gag degradation [64]. Importantly, we showed that the cytoplasmic accumulations of Gag did not form because of endocytosed Gag or viral particles derived from the plasma membrane (Additional file 4: Figure S4). These results suggest that the Staufen1 plays roles in the anterograde transport of Gag, but is also a host factor involved in the formation of the HIV-1 RNP during viral egress and assembly.

## Conclusions

In the present study, we demonstrate that the intermolecular associations between the host mRNA-binding protein Staufen1 and HIV-1 Gag occur in the cytoplasm and at the plasma membrane in HeLa cells and T lymphocytes. Furthermore, we demonstrate that Staufen1 is recruited by Gag to lipid raft microdomains and present results that indicate Staufen1 has potentially novel functions in the intracellular trafficking of HIV-1 Gag during viral egress.

## Methods

### Cell culture and transfections

HeLa cells and Jurkat T cells were maintained under standard conditions at 37°C with in Dulbecco's modified Eagle's medium or RPMI, respectively, supplemented with 10% fetal bovine serum (FBS), 100 units/ml penicillin and 100 mg/ml streptomycin (Invitrogen). Lipofectamine 2000 (Invitrogen) was used for the DNA transfections of Jurkat T cells or HeLa cells according to the manufacturer's protocol. 1 day before transfection 1.5-1.8 × 10^5 ^or 4-5 × 10^5 ^HeLa cells were seeded in 6-well plates or 60-mm dishes, respectively to have ~60% confluency when transfected. 10^6 ^Jurkat T cells were transfected in 25 ml tissue culture flasks.

### BiFC and TriFC analysis

For the BiFC and TriFC experiments, HeLa cells were plated on poly-D-lysine-coated 18 mm micro-glass coverslips (VWR) or 40 mm Bioptechs cover glasses 20-24 hr prior to transfection. Cells were transfected with 1.5 μg to 3 μg plasmid DNA per 18 mm and 3 μg to 5 μg per 40 mm depending on the design of the experiment. Jurkat T cells were transfected in suspension. At 24 hr post-transfection coverslips (HeLa cells) were mounted on a Bioptechs FCS3 imaging perfusion chamber (Bioptechs, Inc.), continuously perfused with fresh medium and warmed using a heating element (Bioptechs, Inc.). For Jurkat T cells, cell adherence to coverslips was promoted using poly-D-lysine (Sigma-Aldrich, Inc). Live cell imaging was performed at 37°C using either Zeiss Pascal LSM5 confocal microscope (Carl-Zeiss, Inc.) with 63×1.3 oil immersion objective or inverted Leica fluorescence microscope. Time-lapse images were captured using 63×1.3 oil immersion objective mounted on a motorized Leica microscope equipped with a PerkinElmer ERS spinning disk confocal system with heated stage and chamber to maintain the cells at 37°C and CO_2_. Images were collected at the times shown or as stated otherwise, for the indicated period. Two-dimensional data sets were deconvoluted using AutoDeblur (MediaCybernetics, Inc.) and compiled using Imaris 6.3.1 software (Bitplane, Inc.). In some experiments, BiFC and TriFC signals were measured in two-dimensional single confocal planes using Imaris software.

### Immunofluorescence and Fluorescence *in situ *hybridization (IF/FISH) analyses

Laser scanning confocal microscopy was performed using a Leica microscope equipped with a PerkinElmer ERS spinning disk or a Carl-Zeiss LSM5 Pascal confocal microscope . Combined 2- and 3-colour IF/FISH co-analyses were performed exactly as described [13]. Images were captured at 512×512 or at 1024×1024 pixels resolution.

### SDS-PAGE, Western blot analysis and antibodies

SDS-PAGE and western blotting were performed as described earlier [49]. Antibodies used included: rabbit anti-Caveolin-1 (Santa Cruz); rabbit anti-Tuberin (TSC1, Abcam); rabbit anti-p24 (Intracell); mouse anti-p24 (NIH); rabbit anti-IMP1 (gift from Finn Nielsen, Rigshospitalet, Copenhagen, Denmark); mouse or rabbit anti-Staufen1 (gifts from Luc DesGroseillers, Université de Montréal, Canada and Graciela Boccaccio, University LeLoir, Argentina); rabbit anti-ABCE1 (a gift from Jais Lingappa, University of Washington); mouse anti-GFP (Roche) and mouse anti-Luciferase (Sigma-Aldrich).

### Lipid rafts staining

To visualize the distribution of insoluble membrane microdomains we used Vybrant Lipid Raft Labeling Kit (Invitrogen) according to the manufacturer's protocol. The method relies on the binding of a red-fluorescent AlexaFluor 594 conjugate of cholera toxin subunit B (CT-B) to the pentasaccharide chain of plasma membrane ganglioside GM1 (a lipid raft marker). An antibody that recognizes CT-B is then used to cross-link the CT-B-labeled lipid rafts into distinct patches on the plasma membrane, which we visualized by microscopy. The coverslips with stained live cells were mounted on Bioptechs FCS3 live cell perfusion chamber and were visualized by laser scanning confocal microscopy.

### Isolation and analysis of detergent-free lipid rafts

We used a simplified method for the fractionation of lipid rafts that does not require the use of detergent [45]. All procedures were carried out with RNAse-free equipment and materials and on ice. Briefly, for each of the cases, two 175 cm^2 ^of HeLa cells were transfected with corresponding plasmids. 24 hr later they were washed 3 times and scraped into buffer B1 (20 mM Tris-HCl pH7.8, 250 mM sucrose, 1 mM CaCl_2_, 1 mM MgCl_2 _and RNAse out (Invitrogen) 1 μl/5 ml), centrifuged for 5 min at 1500 rpm and resuspended again in 1 ml of buffer B1 containing complete protease inhibitor cocktail (Roche). The cells were homogenized in RNAse free 1.5 ml Eppendorf tubes with plastic pestles, centrifuged at 1000 rpm for 10 min and the supernatant (S1) was collected. The pellet was lysed in 1 ml of B1 and homogenized again. Following centrifugation at 500 × *g *for 10 min, the second supernatant (S2) was collected and mixed with S1 (total ~2 ml). 2 ml of 50% OptiPrep (diluted in B1 without Ca^2+ ^and Mg^2+ ^to reach 50%) was added to S1 and S2. The resulting 4 ml of 25% OptiPrep mixture was first poured in 12 ml centrifugation tubes (Beckman Coulter). A step gradient 0-20% was then created using 1.6 ml of 20%, 15%, 10%, 5% and 0% OptiPrep mixtures. The samples were centrifuged in a Beckman ultracentrifuge for 90 min at 52 000 × *g *with rotor SW41. After centrifugation 0.66 ml fractions (18 in total) starting from the top of the tube were collected and 150 μl were used for western blot analysis. Optical densities of resultant bands were quantified using ImageJ software (freeware from the NIH) as described [59].

### Cholesterol depletion

For the complete depletion of cellular cholesterol in live cells, cells were first stained with Vybrant Lipid Raft Labeling Kit (Invitrogen). Coverslips were then mounted in Bioptechs environmental chamber and by perfusion DMEM medium was exchanged with DMEM containing 30 mM 2-hydroxypropyl-β-cyclodextrin (HβCD) [67]. Pictures were taken at *t *= 0 min before addition HβCD and at different time points after addition as indicated in the figures.

### Plasmid expression constructs and siRNAs

The plasmids pMS2-Venus, pMS2-VN, pStaufen1-VC, pIMP1-VC, pFMRP-VC, pGL3-basic/site and pGL3-βActin zipcode have been described previously [33]. To generate pMS2-Staufen1, pMS2-humanized(h)Gag and pMS2-Gag(C36S) vectors Staufen1, hGag and Gag(C36S) sequences were amplified by PCR from pcDNA-Staufen1-TAP [68], pCMV55M1-10 [24] and pNL4.3(C36S) [69] and replaced the VN sequence from pMS2-VN between XhoI and NotI. To generate phGag-VenusC and pGag(C36S)-VenusC, hGag and Gag(C36S) (amplified from pNL4.3(C36S) [69]) PCR fragments replaced IMP1 sequence from pIMP1-VenusC between NheI and XhoI. The Rev-responsive Gag expressor, pSVGagRRE-R was generously supplied by David Rekosh (University of Virginia, U.S.A.; [70]).

For the generation of mRNA reporter expressing plasmids pGL3MS2site-*psi *or pGL3MS2site-Delta-*psi *the fragments containing vRNA packaging signal *psi *(52 bp) and delta *psi *(33 bp) were amplified from plasmids HxBRU and plasmid pHXBAP1 [24] and cloned in pGL3MS2site/basic vector between NheI and XhoI. All plasmids were purified using the Sigma GeneElute Maxi-prep kit. pCMV-Rev was obtained from the National Institutes of Health AIDS Research and Reference Reagent Program. The complete list of plasmids used in this work is given in Table 1.

siRNAs used to deplete Staufen1 (targeting both isoforms - 55 and 63 kDa, siStaufen) or the higher molecular weight isoform (Staufen1-63 kDa) were described earlier [22,49].

## Competing interests

The authors declare that they have no competing interests.

## Authors' contributions

M.P.M. and A.J.M. designed experiments and analyzed data; M.P.M. performed the experiments and drafted the manuscript; C.M.B. provided expression vectors and detailed advice on their use; all authors edited and approved the manuscript.

## Supplementary Material

Additional file 1 **Supplemental Figure S1. Controls for the specificity of the BiFC method**. (A) HeLa cells were transfected with the fusion proteins MS2-VN and Gag-VC (top panels) or with MS2-VN and Staufen1-VC (bottom panels) and examined for green fluorescent signals (i.e., BiFC) at 24 hr post-transfection. (B) HeLa cells were co-transfected with pNL4.3-WT and Gag-VN/Gag-VC (top panels), Gag-VN/Staufen1-VC (middle panels) or Gag-VN/IMP1-VC BiFC pairs (bottom panels). At 24 hr post-transfection, cells were fixed in 4% paraformaldehyde, permeabilized with 0.2% Triton X-100 and processed for fluorescence *in situ *hybridization analyses to identify the vRNA. Black and white renditions are presented for BiFC and vRNA signals with phase contrast and merged colour images in 1^st ^and 4^th ^columns, respectively. The vRNA is shown in red. Hatched areas are magnified in insets on right. The size bars are equal to 10 μm. Click here for file

Additional file 2 **Supplemental Figure S2. Time-lapse confocal imaging of Gag-Staufen1**. HeLa cells were co-transfected with pCMV-Rev, Gag-VN and Staufen1-VC. At 24 hr post-transfection, Gag-Staufen1 BiFC complexes were imaged every 15 sec for a period of 2 min in the same cell. Two-dimensional data sets were analysed using Imaris software (Bitplane, Inc.). The BiFC signals for Gag-Staufen1 BiFC complexes in a selected area (boxed) are presented as a time series from 0 to 120 sec. The movements of the dynamic vesicle-like structures harbouring Gag-Staufen1 BiFC complexes are identified with arrowheads. Blue arrows track the fission events of these structures. The size bars are equal to 10 μm. Click here for file

Additional file 3 **Supplemental Figure S3. Depletion of Staufen1 alters the distributions of Gag-Gag BiFC and *gag *mRNA, but not that of ABCE1**. HeLa cells were co-transfected with pCMV-Rev, Gag-VN and Gag-VC plasmids with either control siNS (A), or siStaufen1 **(B) **or with Staufen1-HA **(C)**. At 24 hr post-transfection the cells were fixed in 4% paraformaldehyde, permeabilized with 0.2% Triton X-100 and processed for combined immunofluorescence and fluorescence *in situ *hybridization analyses to identify ABCE1 (1^st ^column) and the *gag *RNA (2^nd ^column). Gag-Gag BiFC signals (3^rd ^column) were stable following fixation. A merged colour rendition is shown in the 4^th ^column (the *gag *RNA is shown in blue). The size bars are equal to 10 μm. Click here for file

Additional file 4 **Supplemental Figure S4. Depletion of Staufen1 causes cytoplasmic and juxtanuclear redistribution of Gag-Gag multimers when endocytosis is inhibited**. HeLa cells were co-transfected pCMV-Rev, Rev-dependent Gag DNA and the *trans*-dominant negative mutant Eps15-TDN-GFP **(A) **or Rab5-TDN-GFP **(B) **in order to assess the contribution of the endocytosis to the relocalization of Gag (and Gag-Gag BiFC signals) in Staufen1-depleted cells. The cells were fixed at 24 hr post-transfection and processed for immunofluorescence to detect Gag. Cells co-expressing Gag and the TDN-GFP protein were identified. Phase contrast (1^st ^column in **(A) **and **(B)**), black and white renditions for Eps15-TDN-GFP or Rab5-TDN-GFP (2^nd ^column in **(A)**), Gag (3^rd ^column) and colour merges without (4^th ^column) and with (5^th ^column) phase contrast are shown. The size bars are equal to 10 μm. Click here for file

Additional file 5 **Supplemental Figure S5. Tethering of MS2-VenusFL (full length) fusion protein to mRNA reporters used in the study**. mRNA reporter molecules based on pGL3MS2 site/basic harbouring different regulatory protein binding domains - β-Actin zipcode (pGL3-βActin zipcode), HIV-1 vRNA packaging signal *psi *(pGL3MS2site-*psi*) and delta *psi *(pGL3MS2site-Delta-*psi*) were co-expressed in HeLa cells with the fusion MS2-Venus protein. 24 and 40 hr later TriFC signals were visualized in live cells by laser scanning confocal microscopy. Western blotting analyses of Luciferase and MS2-Venus are shown in the bottom panel. The size bars are equal to 10 μm. Click here for file

Additional file 6 **Supplemental Figure S6. Rev-dependent Gag recruits host protein Staufen1 while bound to *psi *RNA**. (A) Modified TriFC system employing Rev-dependent Gags. HeLa cells were co-transfected with pGL3MS2site-*psi*, pCMV-Rev, MS2-VN, and Rev-dependent Gag **(A) **or GagΔNC/p6 **(B)**. Either Gag-VC (Rev-dependent) or Staufen1-VC were included in transfections of **(A) **or **(B)**. TriFC signals were visualized at 24 hr post-transfection by laser scanning confocal microscopy. Gag, expressed from a Rev-dependent expressor in this experiment, can bind *psi *RNA directly or serve as a bridging molecule between the RNA packaging signal, *psi *and either (Rev-dependent) Gag-VC **(C) **or Staufen1-VC **(D)**. The expression of GagΔNC/p6 prevents both the Gag-Staufen1-VC and Gag-*psi *RNA interaction and does not result in TriFC **(B and E)**. The size bars are equal to 10 μm. Click here for file
