# Supplementary figures and images for: Live cell visualization of the interactions between HIV-1 Gag and the cellular RNA-binding protein Staufen1
Source: Retrovirology. 2010 May 10;7:41. doi: 10.1186/1742-4690-7-41 (PMC2887380; doi:10.1186/1742-4690-7-41)

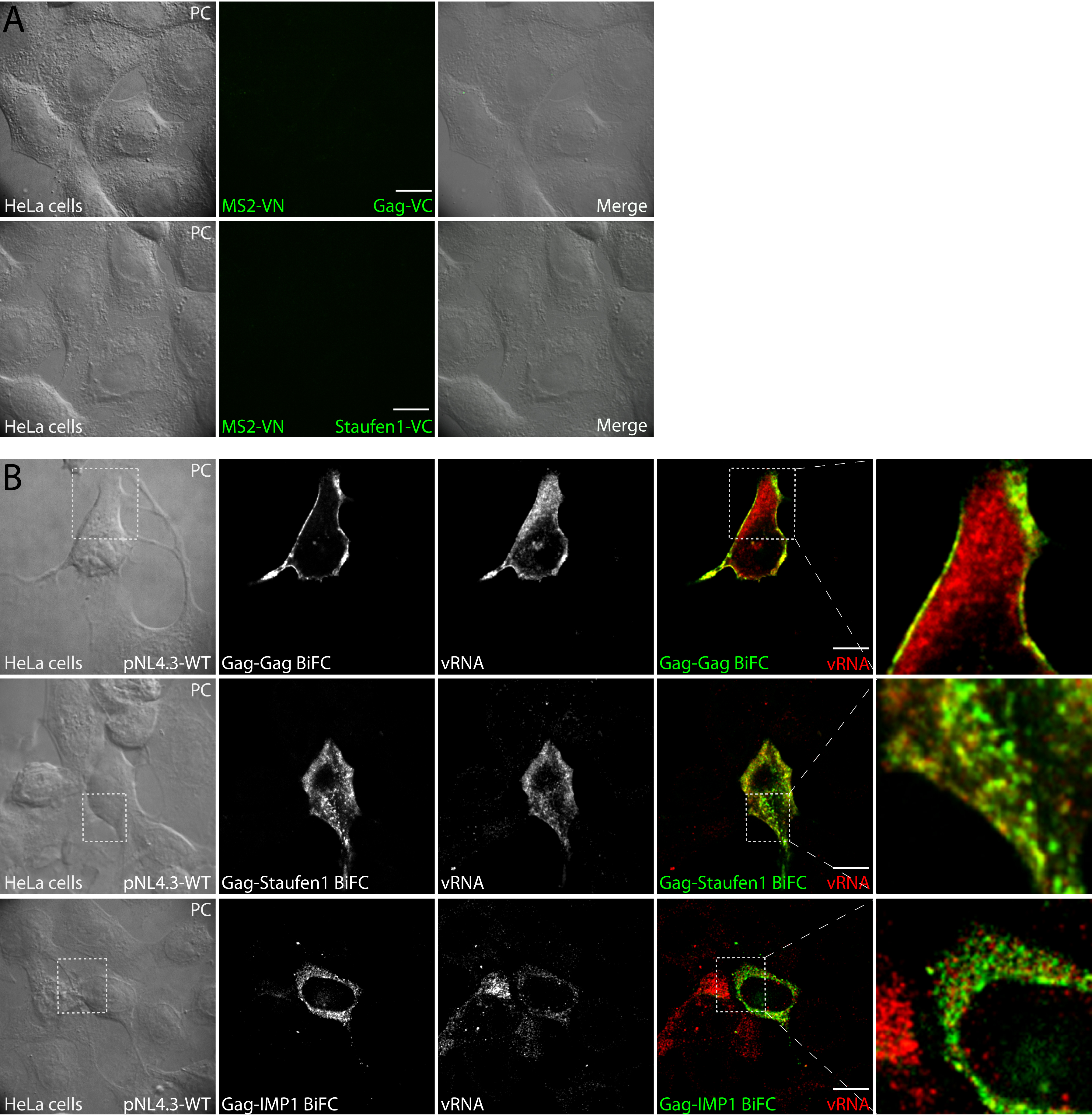

Supplement: Additional file 1 — Supplemental Figure S1. Controls for the specificity of the BiFC method. (A) HeLa cells were transfected with the fusion proteins MS2-VN and Gag-VC (top panels) or with MS2-VN and Staufen1-VC (bottom panels) and examined for green fluorescent signals (i.e., BiFC) at 24 hr post-transfection. (B) HeLa cells were co-transfected with pNL4.3-WT and Gag-VN/Gag-VC (top panels), Gag-VN/Staufen1-VC (middle panels) or Gag-VN/IMP1-VC BiFC pairs (bottom panels). At 24 hr post-transfection, cells were fixed in 4% paraformaldehyde, permeabilized with 0.2% Triton X-100 and processed for fluorescence in situ hybridization analyses to identify the vRNA. Black and white renditions are presented for BiFC and vRNA signals with phase contrast and merged colour images in 1st and 4th columns, respectively. The vRNA is shown in red. Hatched areas are magnified in insets on right. The size bars are equal to 10 μm. [file 1742-4690-7-41-S1.JPEG]

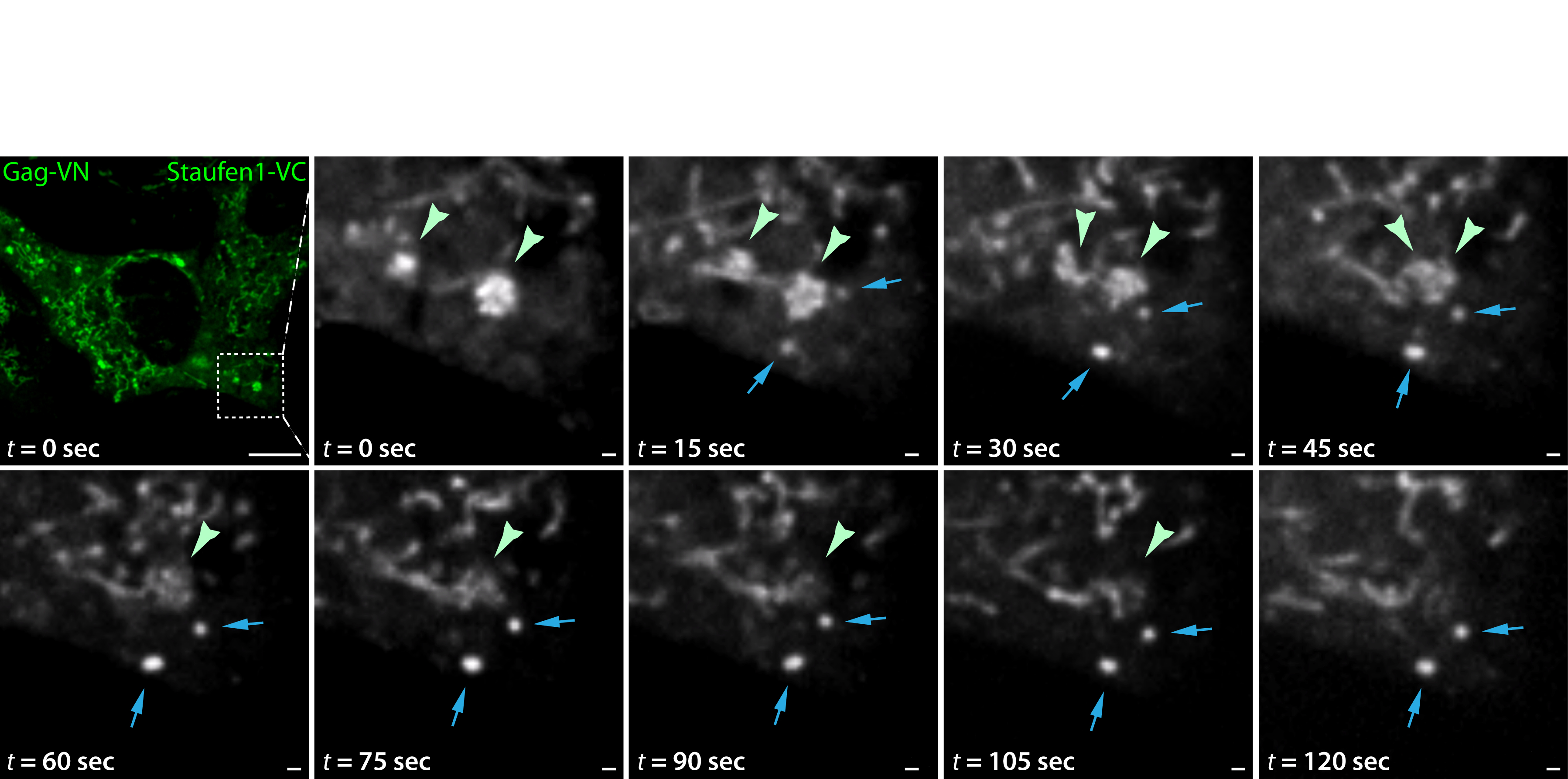

Supplement: Additional file 2 — Supplemental Figure S2. Time-lapse confocal imaging of Gag-Staufen1. HeLa cells were co-transfected with pCMV-Rev, Gag-VN and Staufen1-VC. At 24 hr post-transfection, Gag-Staufen1 BiFC complexes were imaged every 15 sec for a period of 2 min in the same cell. Two-dimensional data sets were analysed using Imaris software (Bitplane, Inc.). The BiFC signals for Gag-Staufen1 BiFC complexes in a selected area (boxed) are presented as a time series from 0 to 120 sec. The movements of the dynamic vesicle-like structures harbouring Gag-Staufen1 BiFC complexes are identified with arrowheads. Blue arrows track the fission events of these structures. The size bars are equal to 10 μm. [file 1742-4690-7-41-S2.JPEG]

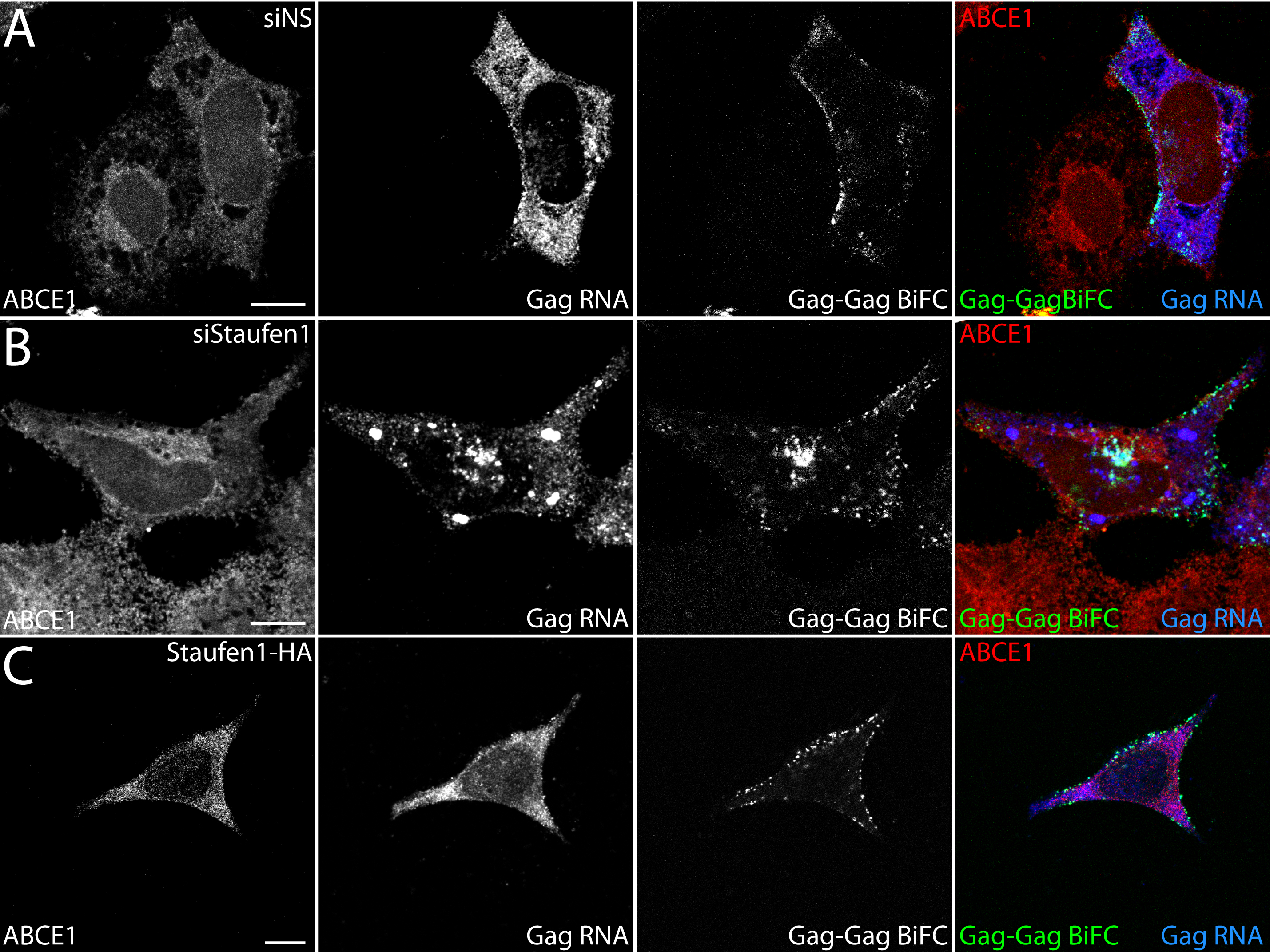

Supplement: Additional file 3 — Supplemental Figure S3. Depletion of Staufen1 alters the distributions of Gag-Gag BiFC and gag mRNA, but not that of ABCE1. HeLa cells were co-transfected with pCMV-Rev, Gag-VN and Gag-VC plasmids with either control siNS (A), or siStaufen1 (B) or with Staufen1-HA (C). At 24 hr post-transfection the cells were fixed in 4% paraformaldehyde, permeabilized with 0.2% Triton X-100 and processed for combined immunofluorescence and fluorescence in situ hybridization analyses to identify ABCE1 (1st column) and the gag RNA (2nd column). Gag-Gag BiFC signals (3rd column) were stable following fixation. A merged colour rendition is shown in the 4th column (the gag RNA is shown in blue). The size bars are equal to 10 μm. [file 1742-4690-7-41-S3.JPEG]

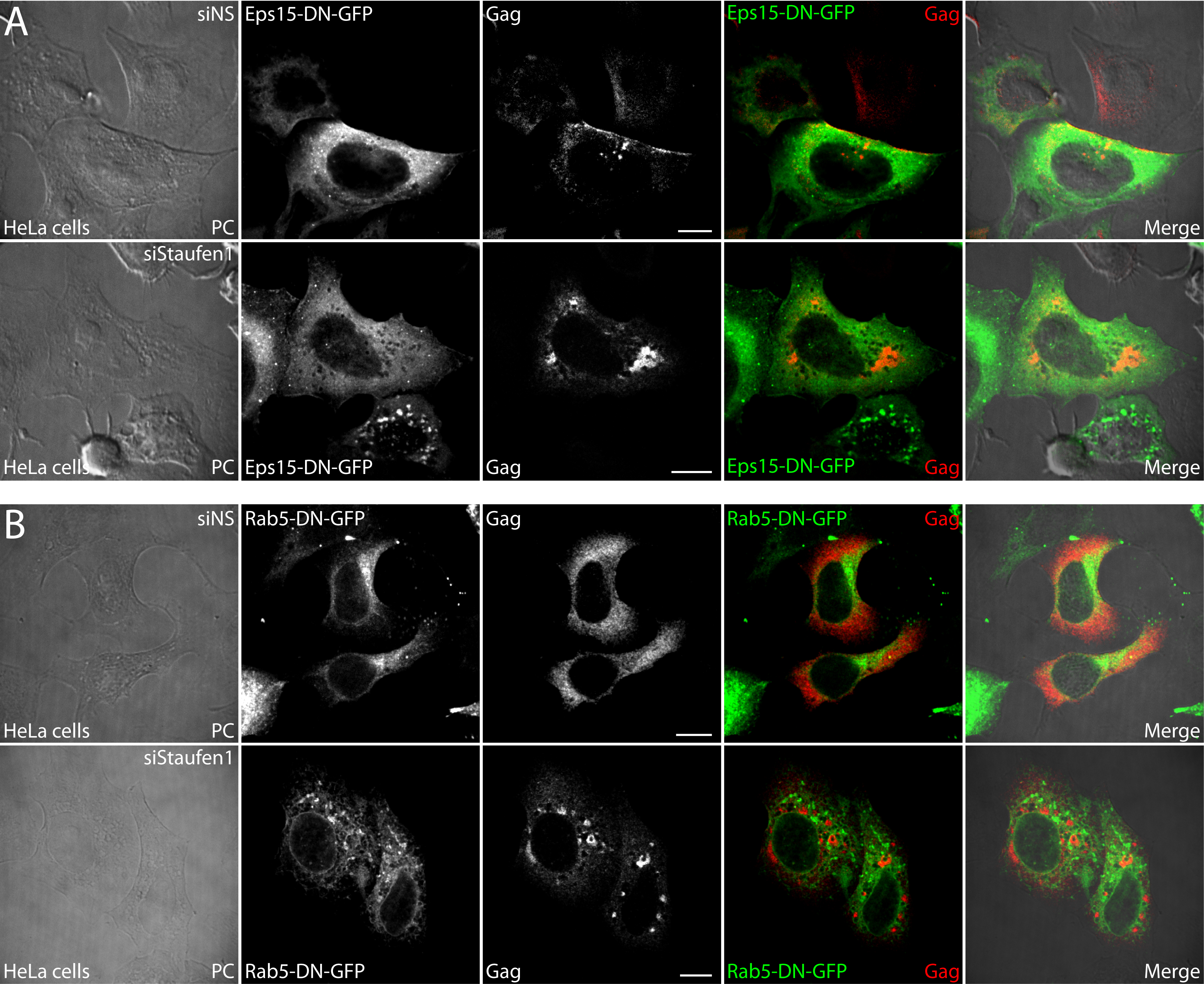

Supplement: Additional file 4 — Supplemental Figure S4. Depletion of Staufen1 causes cytoplasmic and juxtanuclear redistribution of Gag-Gag multimers when endocytosis is inhibited. HeLa cells were co-transfected pCMV-Rev, Rev-dependent Gag DNA and the trans-dominant negative mutant Eps15-TDN-GFP (A) or Rab5-TDN-GFP (B) in order to assess the contribution of the endocytosis to the relocalization of Gag (and Gag-Gag BiFC signals) in Staufen1-depleted cells. The cells were fixed at 24 hr post-transfection and processed for immunofluorescence to detect Gag. Cells co-expressing Gag and the TDN-GFP protein were identified. Phase contrast (1st column in (A) and (B)), black and white renditions for Eps15-TDN-GFP or Rab5-TDN-GFP (2nd column in (A)), Gag (3rd column) and colour merges without (4th column) and with (5th column) phase contrast are shown. The size bars are equal to 10 μm. [file 1742-4690-7-41-S4.JPEG]

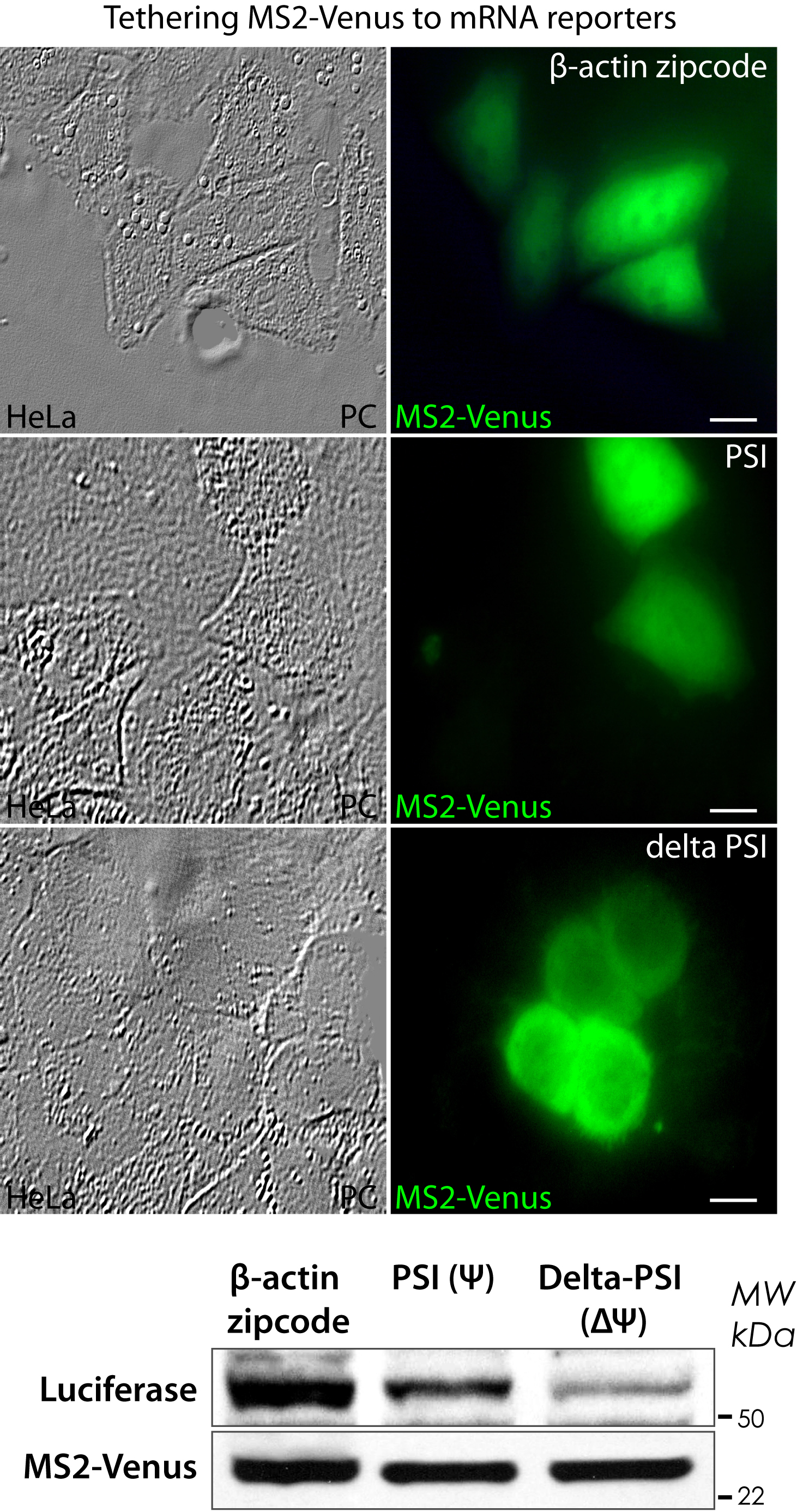

Supplement: Additional file 5 — Supplemental Figure S5. Tethering of MS2-VenusFL (full length) fusion protein to mRNA reporters used in the study. mRNA reporter molecules based on pGL3MS2 site/basic harbouring different regulatory protein binding domains - β-Actin zipcode (pGL3-βActin zipcode), HIV-1 vRNA packaging signal psi (pGL3MS2site-psi) and delta psi (pGL3MS2site-Delta-psi) were co-expressed in HeLa cells with the fusion MS2-Venus protein. 24 and 40 hr later TriFC signals were visualized in live cells by laser scanning confocal microscopy. Western blotting analyses of Luciferase and MS2-Venus are shown in the bottom panel. The size bars are equal to 10 μm. [file 1742-4690-7-41-S5.JPEG]

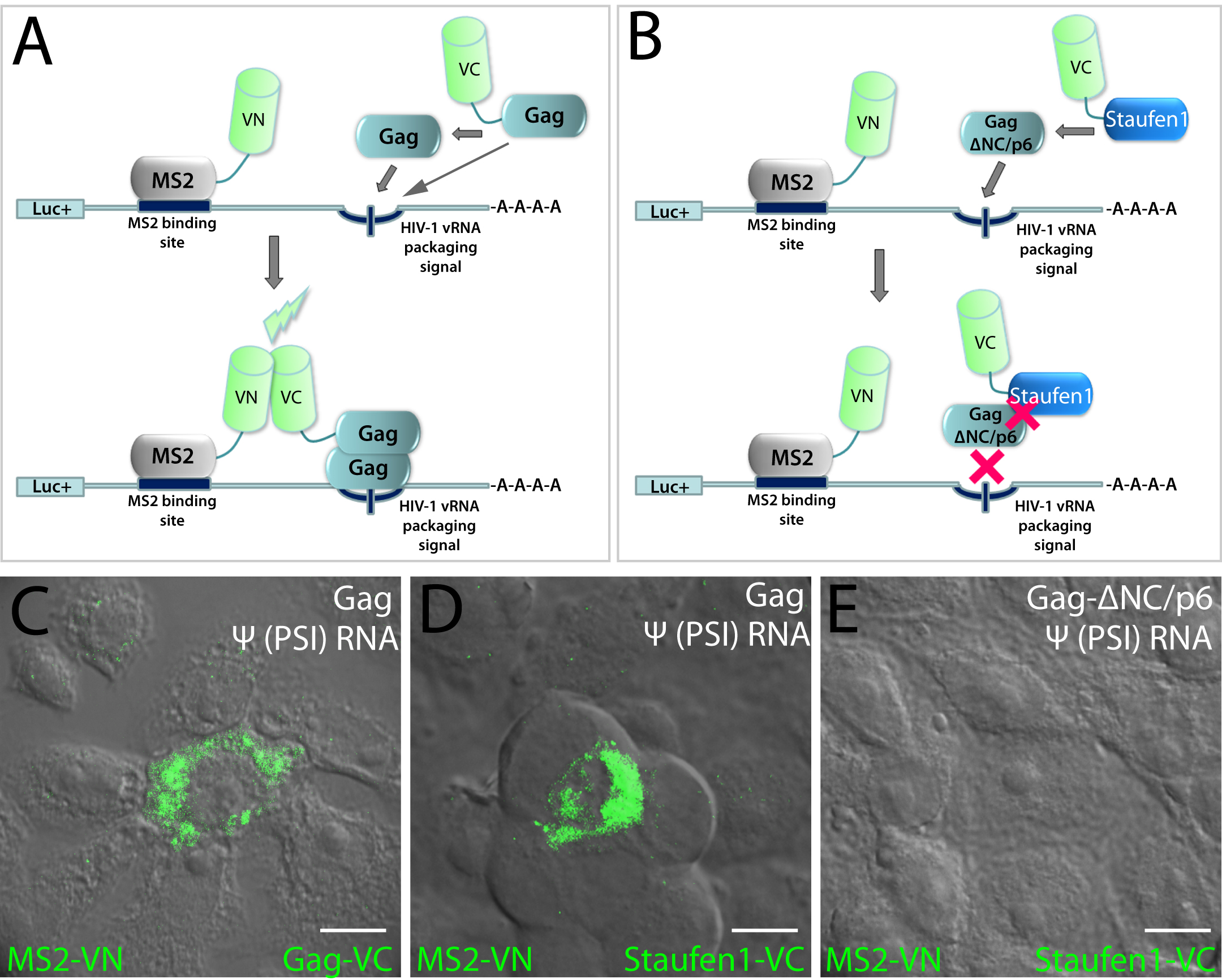

Supplement: Additional file 6 — Supplemental Figure S6. Rev-dependent Gag recruits host protein Staufen1 while bound to psi RNA. (A) Modified TriFC system employing Rev-dependent Gags. HeLa cells were co-transfected with pGL3MS2site-psi, pCMV-Rev, MS2-VN, and Rev-dependent Gag (A) or GagΔNC/p6 (B). Either Gag-VC (Rev-dependent) or Staufen1-VC were included in transfections of (A) or (B). TriFC signals were visualized at 24 hr post-transfection by laser scanning confocal microscopy. Gag, expressed from a Rev-dependent expressor in this experiment, can bind psi RNA directly or serve as a bridging molecule between the RNA packaging signal, psi and either (Rev-dependent) Gag-VC (C) or Staufen1-VC (D). The expression of GagΔNC/p6 prevents both the Gag-Staufen1-VC and Gag-psi RNA interaction and does not result in TriFC (B and E). The size bars are equal to 10 μm. [file 1742-4690-7-41-S6.JPEG]
